# Geographic and demographic gaps in publicly available Alzheimer’s disease datasets: A large language model-based discovery and analysis

**DOI:** 10.1177/20552076261470698

**Published:** 2026-07-21

**Authors:** Mansi Singhal, Joanna Lin, Megan Delehanty, Ali Karimi, Leticia Rittner, Mariana Bento

**Affiliations:** 1 Department of Biomedical Engineering, 2129 University of Calgary, Calgary, AB, Canada; 2 Department of Electrical and Software Engineering, 2129 University of Calgary, Calgary, AB, Canada; 3 Department of Philosophy, 2129 University of Calgary, Calgary, AB, Canada; 4 Department of Communication, 2129 University of Calgary, Calgary, AB, Canada; 5 School of Electrical and Computer Engineering, 28132 University of Campinas, Campinas, Brazil

**Keywords:** data gaps, Alzheimer’s disease, geographical imbalance, demographic representation, large language models, prompt, engineering

## Abstract

**Introduction:**

Alzheimer’s disease (AD) affects millions worldwide, and researchers heavily rely on datasets for diagnosis and treatment. Identifying relevant datasets is challenging due to data gaps and bias related to the demographics and geographic origin.

**Method:**

We investigated AD data gaps by identifying and manually curating publicly accessible AD datasets containing imaging and/or tabular data. We also extracted key information such as data availability, geographic location, and participant demographics. We used five Large Language Models (LLMs) to identify AD datasets, allowing us to explore potential datasets while also evaluating retrieval consistency across models.

**Result:**

We identified 24 publicly accessible AD datasets (open access or controlled access via registration). These datasets enabled us to emphasize three critical gaps: (1) variability in AD dataset retrieval, as observed through differences in LLM outputs, related to dataset visibility and accessibility; (2) geographical imbalance, with North America contributing 55.6% of datasets, US alone 66.7%, followed by Europe at 36.1%, and smaller shares from South America 11.1%, Asia 8.3%, and Africa 2.8%; and (3) demographic deficits, with the majority of datasets predominantly White, as 9 of 24 had over 80% White participants. Among the seven datasets that reported any Black participant representation, the proportion of Black participants ranged from 15.3% to 18.8%.

**Conclusion:**

These findings reveal significant disparities in the availability and retrieval of AD datasets, with most data concentrated in Western countries and critical gaps in demographic representation. LLMs show inconsistent retrieval, particularly for newer, smaller, or region-specific datasets, which may perpetuate existing biases.

## Introduction

Dementia affects millions of people worldwide, with 10 million new cases every year, and the most common form of dementia, Alzheimer’s Disease (AD), contributes to nearly 60-70% of cases.^1^ Research on AD shows that the biggest challenge is finding a suitable dataset, but sometimes those datasets are an issue because they are incomplete and hard to access. One of the most widely used datasets is the Alzheimer’s Disease Neuroimaging Initiative (ADNI) database. While it includes a large number of participants, it also suffers from a high rate of missing features. The lack of sufficient data leads to challenges in AD research, and access to more AD datasets with less bias and missing information is the utmost requirement.^2^

The recent report on Alzheimer’s Disease International^3^ provides information on misconceptions about AD. Almost 80% of the general public and 65% of health care professionals still believe that dementia is part of normal aging. This misconception is particularly prevalent in low- and middle-income countries (LMICs),^4^ with more than 61% of people in high- and middle-income countries believing AD is related to an unhealthy lifestyle. These attitudes contribute to social exclusion, with 88% of people living with dementia reporting experiences of discrimination. In 2025, AD rates by country based on the World Population Review^5^ show that Japan has the highest prevalence of AD, with 2,710 cases per 100,000 people, followed by Germany, Italy, Greece and Monaco, all with high rates driven primarily by aging populations. On the other hand, regions such as Africa and Southeast Asia have fewer reported cases, though these figures may be undervalued due to limited diagnostic frameworks and inherent underreporting. Within countries, prevalence can also be modulated by rural vs. urban residence, as well as social determinants of health, such as access to care and educational opportunities. However, according to the world health organization over 60% of people suffering from AD live in low- and middle-income countries, according to the World Health Organization.^1^ Therefore, based on this analysis, there is a disparity in diversity across geography, which leads to the disease burden and lack of AD research representation around the world. This geographic bias has epistemic consequences. AI models are trained on data from Western, educated, industrialized, rich, and democratic (WEIRD) countries.^6^

Every population is not receiving equitable treatment because of racial and ethnic disparities,^7^ Lim et al.^8^ studies on population show that Asian Americans, Native Hawaiians/Pacific Islanders, American Indians/Alaska Natives, and multiracial people are underrepresented in AD research cohorts, despite having variable risk. Risk factors associated with these disparities include both modifiable elements and non-modifiable genetic factors.^9^ These factors are affected by local environments and access to health systems. Geographic and ethnic gaps in research participation result in epistemic limitations. The ethical challenges include the health inequities and the loss of opportunities for culturally modified approaches.^10^ Alzheimer’s Disease International^3^ has emphasized the need for better data collection in diverse settings and to include majority populations in research. The work shows that until these disparities are addressed, scientific advances may continue to miss critical opportunities for inclusive understanding, precision medicine, and reducing the global burden of AD.

Most AD datasets are derived from high-income countries and substantially represent White populations, while individuals from low- and middle-income regions and diverse ethnic backgrounds persist underrepresented. This lack of diversity results in significant literature gaps, including a limited understanding of how AD presents and progresses between different populations, and restricts the advancement of diagnostic treatments that are effective for all groups. Investigating data gaps is therefore essential to ensure that advances in AD research and care are equitable and benefit the global population.

In this study, we investigate AD data gaps by identifying and manually curating publicly accessible AD datasets using five large language models (LLMs). The main contribution of this work is threefold:

(1) a curated, manually verified inventory of 24 publicly accessible AD datasets with extracted geographic and demographic metadata;
(2) a quantitative evaluation of retrieval consistency across five LLMs (ChatGPT, DeepSeek, Perplexity AI, Google Gemini, and Microsoft Copilot) using precision, recall, and F1 scores; and
(3) an analysis of geographic and demographic gaps in these datasets, including a novel framework describing the ‘visibility bias reinforcing cycle’ whereby LLMs perpetuate existing dataset inequities.

This study differs from existing reviews of AD datasets e.g., Borchert et al.^11^ and health data bias literature in three key ways. First, while prior reviews have documented overreliance on ADNI, we provide the first systematic evaluation of LLMs as tools for AD dataset discovery. Second, unlike descriptive dataset surveys, we explicitly quantify LLM retrieval bias and demonstrate its consequences for dataset visibility. Third, we show that the problem is not merely one of data availability but of a feedback loop where LLMs systematically direct researchers toward already-dominant datasets, marginalizing diverse resources. By making these contributions explicit, we aim to inform researchers, dataset curators, and LLM developers about the risks of uncritical LLM use in scientific dataset discovery.

To contextualize our investigation of AD data gaps and LLM-based dataset discovery, we next review the existing literature on geographic and demographic biases in AD research, dataset availability challenges, and the emerging role of AI and LLMs in biomedical data retrieval. This review synthesizes prior findings to identify the specific gaps that our study aims to address and to establish the rationale for our methodological approach.

## Literature review

In the literature review, we found that Borchert et al.^11^ analyzed 255 studies that used AI with neuroimaging to look at brain diseases. They observed a strong dataset bias: about 71% of studies used the ADNI dataset, whereas no other dataset was used more than five times. This paper summarizes the requirement of better reporting and bias monitoring to move the AD research to more diverse datasets and not to rely solely on the ADNI dataset.

Zhang et al.^12^ analyzed genome-wide association studies (GWAS) in AD, they found 1,350 articles. The United States (US) was doing most of the work, with 775 publications contributing to the majority of the citations. Things like genes related to immunity, such as DNA methylation, and amyloid-*β*/tau pathology. The analysis pointed to underrepresentation in GWAS involving non-European populations that emphasizes the critical gaps in population diversity.

Further, Moguilner et al.^13^ developed brain clocks using resting-state functional MRI data from over 5,300 participants across 15 countries, including underrepresented populations from Latin America and Africa. Their study revealed that brain age gaps, the difference between predicted brain age and chronological age, were significantly larger in Latin American and African cohorts compared to European cohorts, even after controlling for socioeconomic factors. This finding demonstrates that regional and demographic disparities directly impact the performance of neuroimaging-based biomarkers, reinforcing the concerns raised in our study about geographic bias in AD datasets. Furthermore, the authors emphasize that models trained predominantly on WEIRD populations may fail to generalize across diverse global populations, leading to inaccurate diagnoses and perpetuating health inequities. Their work highlights the urgent need for geographically diverse datasets, precisely the kind of datasets that our LLM-based discovery process found to be critically underrepresented in publicly available AD resources.

Another study, Chan et al.^14^ investigated disparities in referral sources to Alzheimer’s Disease Research Centers across different racial and ethnic groups. The study found that Black, Asian, Hispanic, and other minority groups were underrepresented compared to White participants, largely due to differences in referral pathways and healthcare access. This highlights that referral bias is a systemic issue that must be addressed to ensure equitable participation in AD research. Ethical problems related to data sharing are discussed, particularly in medicine.^15^ When data is shared between different systems, technical and moral issues become more significant. They state that being able to use data across systems is crucial for working together on research in new ways. But it can also cause ethical problems that are different from the ones we see when we are processing data with algorithms or sharing it.

In a 2025 study, Shan et al.^16^ examined potential bias in LLMs. They wanted to see if the models favored certain values. They split it into two parts: first, seeing if the models liked collectivist or individualist ideas, and second, if they even knew the difference between the two. Turns out, some models knew the difference but still had their own biases, while others just did not get it at all. This means just because a model understands something, it does not mean it is neutral. This work provides information on the AI models that have to be accurate and neutral in the way they were created. The study states that for language models to be trustworthy, they have to provide accurate information and not push any specific ideology. This lines up with what we already know, models usually pick up the cultural and political views from the data they are trained on.

A seminal work by Obermeyer et al.^17^ demonstrated algorithmic bias in healthcare, showing that a widely used clinical algorithm systematically underestimated the health needs of Black patients compared to White patients with the same level of illness. This occurred because the algorithm used past healthcare costs as a proxy for health needs, reflecting systemic inequalities in access to care rather than true differences in illness burden. This work provides direct empirical evidence that AI systems trained on biased data can perpetuate and amplify existing health disparities, which aligns with our findings on demographic gaps in AD datasets.

Jones et al.^18^ examined LLMs by combining the ideas from language study and cultural anthropology. This paper gives the information regarding how these LLMs understand and use language connected to different cultures, which they call cultural interpretability. The idea is about the language and culture as they are deeply connected and change depending on the context, which LLMs should reflect to communicate better with people from diverse backgrounds.

Highly cited articles are generally recognized as important in their disciplines.^19^ Following retrieval, it was discovered that all of the literature’s top 100 citations were from highly cited works. In order to accurately reflect the most fundamental and significant advancements in this field of study, this bibliometric analysis included the top 100 citations in the literature. Researchers from the United States, China, the United Kingdom, and other major economies contributed the most, and the University of North Carolina produced the most papers. The diagnosis of AD in the context of AI has produced some useful outcomes with the advancement of medical AI. AD has been predicted and diagnosed in recent years using a range of AI technologies, including machine learning and deep learning. Due to uneven data quality, short cohort sizes, and demographic biases in training datasets, current models have limited generalizability.

Lock et al.^20^ examined the ethical challenges of using neuroimaging data repositories to develop AI models for neurological diseases. They found that most of these datasets come from high-income countries, which raises concerns about fairness and generalizability. Machine learning models trained on these datasets often perform poorly in women and ethnic minorities, potentially worsening healthcare disparities. To demonstrate this problem, the authors showed that changing the composition of a dataset can alter the results of AI models. They argue for creating more balanced repositories, such as one in Southeast Asia, to address these gaps. This work supports our study’s findings that geographic and demographic biases in AD datasets can undermine the reliability and fairness of AI-driven research.

The application of explainable artificial intelligence (XAI) in neurodegenerative disorders has gained significant attention, as it addresses the critical need for transparency and interpretability in AI driven diagnostic systems.^21^ XAI methods help clinicians understand why a model makes a particular prediction, which is especially important in AD where treatment decisions have profound implications for patients. A holistic study of XAI applications in neurodegenerative disorders highlights how explainability can bridge the gap between black box AI models and clinical practice, fostering trust and adoption among healthcare professionals.^21^ Wang et al.^21^ conducted a holistic study of XAI applications across neurodegenerative disorders including AD, Parkinson’s disease, and Multiple Sclerosis, organizing interpretability techniques into distinct categories and identifying research gaps in security frameworks and clinical deployment. Their findings demonstrate that explainability serves as a critical link between opaque AI systems and real-world clinical application, enhancing clinician trust and encouraging adoption, yet they also underscore that the majority of XAI research has yet to progress beyond the experimental phase into routine practice.

The principles of explainable AI extend beyond neurodegenerative diseases to other medical domains. Research on leveraging XAI for transparent and trustworthy cancer detection systems has demonstrated that explainability not only improves model transparency but also helps identify potential biases and errors in model predictions.^22^ These insights are directly transferable to AD research, where similar XAI frameworks can be adopted to ensure that diagnostic models are both accurate and interpretable across diverse patient populations.^23^

A systematic literature review of deep learning applications in AD has synthesized current trends, methodologies, challenges, innovations, and future directions in the field.^24^ Toumaj et al.^25^ classified deep learning applications in AD into four major categories: NLP, drug reuse, classification, and identification, and critically evaluated the explainability and security concerns that limit clinical translation. Their review highlights that while deep learning, particularly CNNs, has achieved remarkable success in AD diagnosis using neuroimaging data, significant challenges remain regarding data standardization, model reproducibility, and generalizability across different populations and imaging protocols. The review also identifies emerging innovations such as multimodal fusion architectures and transfer learning, which align with the data integration challenges discussed in our study.

Established repositories that catalog and provide access to multimodal AD datasets include the National Alzheimer’s Coordinating Center (NACC),^26^ the Laboratory of Neuro Imaging Image Data Archive (LONI IDA),^27^ the Alzheimer’s Disease Data Initiative (ADDI) AD Workbench,^28^ the Scalable Neuroimaging Analytics Toolkit (SCAN), and OpenNeuro.^29^ Additionally, diverse, equity-focused cohort studies such as the Health and Aging Brain Study Health Disparities (HABS-HD),^30^ the ADRC Consortium for Clarity in ADRD Research Through Imaging (CLARiTI),^31^ and Bio-Hermes-001^32^ represent important efforts to address representational gaps, though many of these were not retrieved by the LLMs in our study.

The intersection of distributed computing and medical data processing is critical for managing these diverse data sources. Aminizadeh et al.^33^ comprehensively reviewed ML applications in medical data processing based on distributed systems and IoT, highlighting how these technologies enable efficient processing of large-scale healthcare data while also introducing challenges in data quality, heterogeneity, and real-time analysis issues that are particularly relevant to the multimodal AD datasets discussed above.

The challenge of complex pattern recognition in interconnected information systems is highly relevant to AD research, where data from multiple modalities (imaging, genetics, clinical assessments) must be integrated to capture the multifaceted nature of the disease.^34^ Autonomous learning techniques for complex pattern recognition offer promising approaches for identifying subtle disease signatures across heterogeneous data sources. However, as demonstrated in our study, the effectiveness of these techniques depends critically on the diversity and representativeness of the training data, reinforcing the need for geographically and demographically balanced datasets.

Aminizadeh et al.^35^ further examined the opportunities and challenges of AI and distributed systems in healthcare, finding that IoT platforms (44.3%) and disease diagnosis applications (47.8%) dominate the current landscape. Their review identified critical barriers to data utilization, including interoperability issues and data privacy concerns, which directly parallel the dataset accessibility challenges we identify in our study.

In our research paper, we showed some trends: (1) a heavy reliance on Western-centric datasets, especially ADNI; and (2) the requirement for more diverse data to enhance generalizability. The primary goal of this research article is to identify a list of publicly available AD datasets from around the world using LLMs. Since our study employed LLMs, we could not rely directly on their outputs; therefore, we manually curated and verified the consistency of the datasets. We searched for AD datasets, including both imaging and tabular data types. We aggregated key information from these datasets, such as source, availability, location, and participant demographics, such as age, sex, gender, and ethnicity. We also analyzed critical gaps in dataset representation, particularly geographical imbalances and demographic deficits. Based on our findings, there is a lack of data available for specific populations, especially Black/African American, Hispanic/Latino, Indigenous, and other underrepresented populations. Through this research, we demonstrate how the scarcity of data for equitydeserving populations confines the ability to understand disease progression, risk factors, and treatment responses across diverse groups.

The literature reveals three consistent themes: (1) a heavy reliance on Western-centric datasets, particularly ADNI, in AD research; (2) persistent demographic underrepresentation of non-White populations in research cohorts; and (3) emerging evidence of bias in AI and LLM systems trained on non-representative data. However, no study to date has systematically evaluated LLMs as tools for AD dataset discovery or quantified the relationship between LLM retrieval patterns and underlying dataset inequities. To address this gap, we developed a methodology that combines LLM-based dataset discovery with systematic manual curation and quantitative evaluation of retrieval consistency, geographic distribution, and demographic representation across five major LLM platforms.

## Methodology

### LLM selection

For our methodology, we worked with five LLMs, such as ChatGPT (GPT-4),^36^ DeepSeek (DeepSeek-V3),^37^ Perplexity AI (Perplexity Pro default model),^38^ Microsoft Copilot (Copilot, balanced creativity mode, default model),^39^ and Google Gemini (Gemini 1.5 Pro, free tier),^40^ to find tabular and imaging datasets for AD.

The five LLMs were selected based on three criteria: (1) public availability at the time of study (March–April 2025), (2) distinct underlying architectures or training approaches to maximize variability in retrieval behavior, and (3) common usage in biomedical research contexts based on citation rates and community adoption. ChatGPT (GPT-4) was included as a widely used general-purpose LLM. DeepSeek-V3 was included for its reported strengths in structured information retrieval. Perplexity AI was included for its integration of web search with LLM generation. Google Gemini 1.5 Pro was included as a representative of search-native models. Microsoft Copilot was included for its integration with Microsoft’s search ecosystem. We did not evaluate paid tiers or enterprise versions of these models.

**Figure 1.**
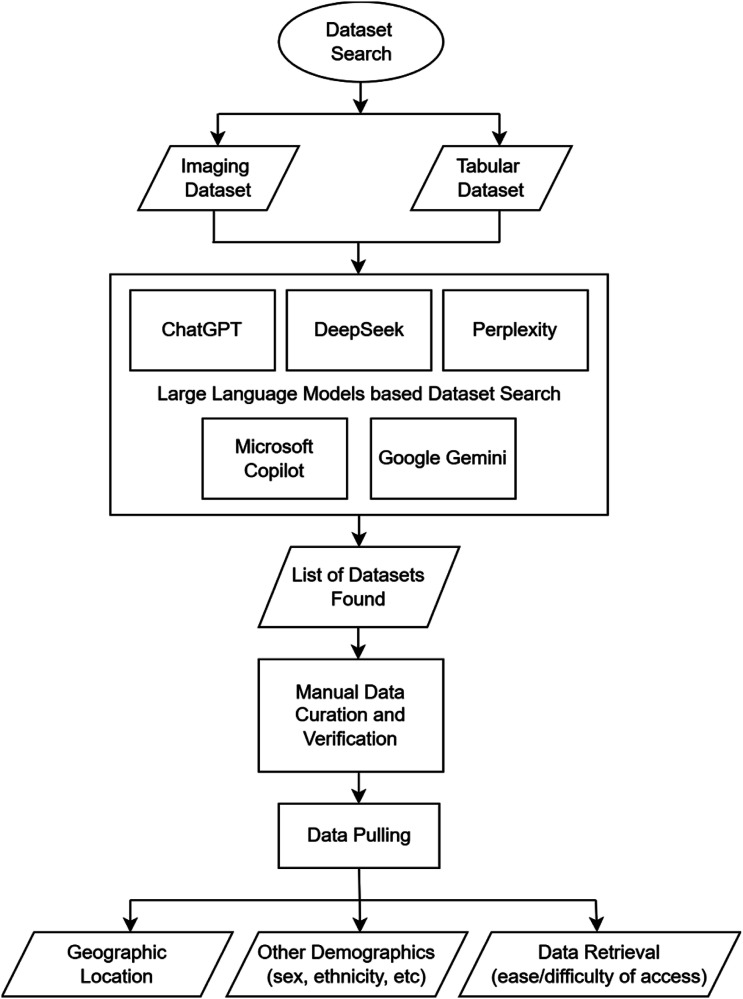
Comprehensive pipeline for imaging and tabular dataset search using LLMs from initial dataset identification to manual curation, categorization, organization, and demographic information extraction.

This gave us a first list of datasets that anyone can access. We then checked these datasets ourselves to make sure that the information was reliable and related to what we were doing. We also extracted important details, such as where to find the data and information about the participants in the datasets, such as their age, sex, and ethnicity. Figure 1 shows the steps of our framework.

### Dataset search strategy and prompt engineering

We asked each LLM the same questions three times, with different prompting each time by using particular keywords and phrases. We kept asking until we reached a point where further searching on LLMs did not give us new results, which we called the diminishing returns in qualitative research, Saunders et al.^41^ We conducted three search rounds per LLM. After each round, we recorded all unique dataset names manually. We defined diminishing returns as two consecutive rounds that produced no new unique datasets. For DeepSeek, saturation was reached after three rounds. For all other LLMs, saturation was reached after two rounds. In this way, we made sure that we asked for enough different AD datasets. We stopped only when they repeatedly returned the same datasets. To be fair, we used a fresh email we had to log in with, so past searches would not create any search bias. In addition, we kept track of when we did all the searches. Our search strategy separately queried imaging and tabular datasets. And used the same search prompt engineering for both of them with LLMs, as described below. However, the analysis presented in this paper does not include modality-specific comparisons (e.g., structural MRI vs. PET vs. clinical data). The results focus exclusively on geographic distribution and demographic representation. No claims are made about modality-specific gaps.

We used a three-tier prompting strategy to vary how specific and constrained each query was. The first prompt was highly structured (e.g., number of subjects, demographics, access status, etc.). This was designed to test how well LLMs could retrieve accurate and complete dataset information when guided by specific metadata requirements. The second prompt removed these constraints and used a more open-ended format to encourage broader retrieval and identify datasets that might be missed under the stricter instructions. The third prompt kept this open-ended format but added a geographic specification (“from around the world”) to assess whether models showed regional bias. This approach allowed us to evaluate the depth of metadata the LLMs could provide and the breadth of their dataset coverage. The same three-tier strategy was applied to both imaging and tabular dataset searches to ensure consistency.

#### Imaging dataset

These datasets contain neuroimaging data that help visualize brain structures, detect atrophy, and find biomarkers like amyloid plaques in AD studies. For this kind of data type, we used three prompts to search on LLMs. The first prompt was, “Provide me with a table containing all the imaging datasets related to Alzheimer’s disease/dementia. Include details about each dataset, such as a description of the dataset, number of subjects, imaging modality details, preprocessing, age range, sex, gender, ethnicity, location, open access status, whether it is paid or free, linkage datasets consortium or a larger initiative, and the dataset link.” The second prompt was, “Give me all the datasets related to Alzheimer’s disease/dementia imaging data as much as you can.” And the last, third prompt was, “Provide me with all available datasets related to Alzheimer’s disease/dementia imaging data from around the world.” For all these searches, the date of search was 30 March 2025.

#### Tabular dataset

Alzheimer’s research uses tabular datasets with patient information, including demographic variables such as age, sex, gender, ethnicity, and location. These datasets also include a wide range of clinical assessments, such as cognitive scales, diagnostic classifications, results from neuropsychological tests, and information on comorbidities and medical history. These also have biomarker measurements, genetic information, medication records, and longitudinal follow-up data. Collectively, these tabular datasets enable a comprehensive analysis of factors related to disease risk, progression, and patient outcomes. Similarly, as we used the LLM for the imaging dataset, we searched with the same prompts for the tabular dataset; three prompts were used. The first prompt was, “Provide me with a table containing all the datasets related to Alzheimer’s disease/dementia tabular datatype. Include details about each dataset, such as a description of the dataset, number of subjects, data types, preprocessing, age range, sex, gender, ethnicity, location, open access status, whether it is paid or free, and dataset link.” The second prompt was, “Give me all the datasets related to Alzheimer’s disease/dementia tabular data as much as you can.” The third prompt was, “Provide me with all available datasets related to Alzheimer’s disease/dementia tabular data from around the world.” The date of search for all the tabular datasets was 20 April 2025.

For each LLM, we conducted three search rounds. A round consisted of submitting all three prompts (structured, open-ended, and open-ended with geographic specification) for both imaging and tabular data types sequentially. Thus, each LLM received a total of 6 prompt submissions per round (3 prompts × 2 data types). Over three rounds, each LLM received 18 prompt submissions. We defined ’diminishing returns’ (saturation) as two consecutive rounds that produced no new unique dataset names after manual deduplication. For DeepSeek, saturation was reached after three rounds; for all other LLMs, saturation was reached after two rounds. All searches were conducted on a single day per model to minimize temporal variability in model updates.

The imaging and tabular searches were conducted approximately three weeks apart (March 30 to April 20, 2025). LLMs are updated continuously, sometimes without public notification. This temporal gap may introduce inconsistency, as model knowledge or behavior could have changed between searches. This limitation is discussed further in the Limitations section.

### Evaluation metrics for LLM retrieval performance

To quantitatively evaluate the retrieval performance of each LLM, we adopted standard information retrieval metrics using our final curated set of 24 AD datasets as the reference standard (“gold standard”). The following definitions were applied:

• True Positive (TP): The LLM correctly identified a dataset from the gold standard list (indicated by a score of 1, 2, or 3 in our retrieval coding).
• False Negative (FN): The LLM failed to identify a dataset that exists in the gold standard list (score of 0).
• False Positive (FP): The LLM suggested a dataset that does not actually exist or is irrelevant to AD (hallucination). These were identified during manual curation and excluded from the final dataset list.

Based on these definitions, we computed:

• Recall (Sensitivity) = TP/(TP + FN) = TP/24
(proportion of gold standard datasets successfully retrieved)
• Precision (Positive Predictive Value) = TP/(TP + FP) (proportion of retrieved datasets that were relevant)
• F1 Score = 2 × (Precision × Recall)/(Precision + Recall) (harmonic mean of precision and recall)

For statistical comparison across LLMs, we conducted a Cochran’s Q test for multiple related samples, followed by pairwise McNemar tests with Bonferroni correction for multiple comparisons (adjusted significance threshold *α* = 0.005). To determine whether LLM-based identification reflects true dataset usage patterns or is influenced by retrieval bias, we also did a comparison against findings from Borchert et al.^11^

### Manual data curation and verification

First, we identified the total number of existing AD datasets available through LLM searches. After finalizing, we proceeded to extract key features of these datasets to assess their relevance and completeness.

We manually reviewed each dataset entry to check the accuracy of the information provided by the LLMs. We started this process by following the dataset link that was provided by the LLMs to verify that it correctly corresponded to the dataset being shown while doing the prompt engineering. If the link matched, we continued to validate the remaining fields by consulting the dataset’s official webpage, documentation from related academic publications, or any associated data platforms that contain the dataset. In many cases, information was sourced from other areas to confirm dataset characteristics without requiring data download.

**Figure 2.**
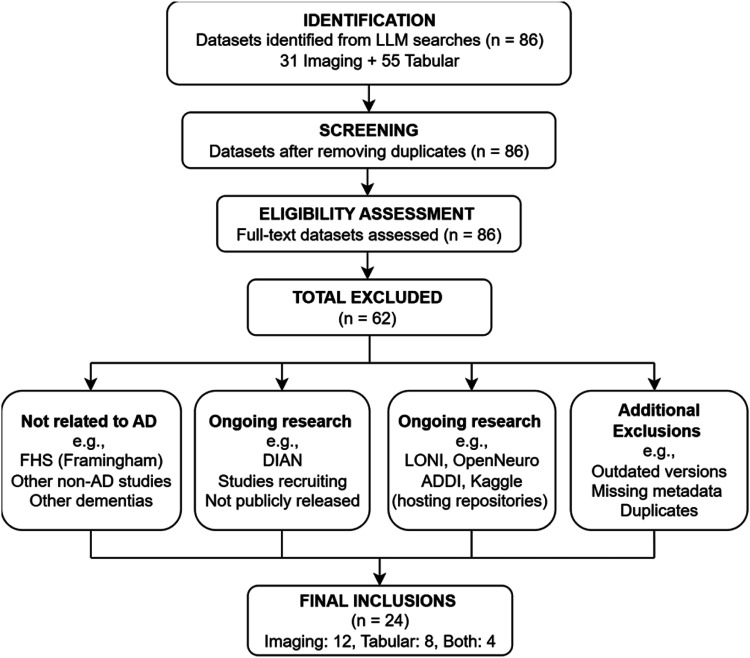
PRISMA flow diagram showing systematic identification, screening, exclusion, and final inclusion of 24 Alzheimer’s disease datasets.

If the LLM provided accurate information, it was left unchanged. If discrepancies were identified, the incorrect value was flagged. A correct version was added along with explanatory notes that included links to the verified information and the rationale for the correction. After checking everything, we removed any datasets that were not related directly to AD. For instance, datasets intended for other conditions, such as FHS (Framingham Heart Study), were excluded unless there was clear evidence of their relevance to AD studies. We also excluded any datasets that were still in progress, such as DIAN (Dominantly Inherited Alzheimer Network), as well as those that had updated versions available, such as AddNeuroMed, as shown in the PRISMA flow diagram in Figure 2.

Datasets that were only hosted on a platform but not produced by the platform were also excluded. Platforms are hosting repositories that provide access to datasets, whereas the actual resources are those that generated the data.

For the purpose of this study, ’publicly accessible’ was defined using a two-tier classification:

Tier 1 – Open Access: Datasets that can be downloaded immediately without any registration, login, or data use agreement. Examples include OASIS and MIRIAD.
Tier 2 – Controlled Access with Registration: Datasets that require users to complete a registration process, agree to data use terms, and potentially undergo approval before accessing data. Examples include ADNI, NACC, and UK Biobank.

We did not exclude datasets based on practical accessibility barriers (e.g., registration burden, approval time, data-use agreements, cost, institutional restrictions, or download limitations), as systematically assessing these factors was beyond the scope of this study. However, we acknowledge that Tier 2 datasets vary substantially in real-world accessibility. For instance, some controlled-access datasets approve all legitimate researchers within days, while others require months of review, letters of intent, or institutional signatures. Some datasets charge fees (e.g., UK Biobank requires a modest access fee), while others are free. Some restrict access to researchers from specific countries or institutions. These practical barriers are noted in the supplemental table where information was available, but a formal assessment was not conducted. Researchers should verify current access requirements before planning studies that depend on Tier 2 datasets.

### Data pulling

After following the initial dataset identification via LLM queries, we did the data pulling and the information extraction procedure was implemented for each dataset. This process was designed to capture a standardized set of metadata essential for analyzing gaps in availability and representation. We extracted the specific information for every dataset, including its official name and acronym, sourced from individual organizations and consortia, and accessibility status, such as open access or permission required. We collected the primary data modalities by categorizing them as either imaging, such as structural MRI, PET, and tabular for e.g., clinical, cognitive, and genetic. To assess geographic and demographic representation, we extracted the dataset’s geographic location and all available participant demographics, including age range, sex, gender, where it is reported, and detailed ethnicity/race composition. This structured extraction enabled the aggregation of key variables into a unified framework for subsequent gap analysis.

Following the systematic search, manual curation, and demographic data extraction procedures described above, we now present our findings. Our analysis yielded a curated set of 24 publicly accessible AD datasets, which we evaluate across three dimensions: (1) the consistency and variability of LLM retrieval performance across the five models, (2) the geographic distribution of datasets and the resulting regional imbalances, and (3) the demographic composition of study participants and the deficits in representation of diverse populations. These findings are presented sequentially below.

## Findings

From the initial list of datasets, we manually curated a final selection of 24 datasets that met our inclusion criteria as outlined in Table 1.

**Table 1.** Availability of key demographic metadata across datasets.

Dataset	Age	Sex	Gender	Ethnicity
**OASIS** (Open Access Series of Imaging Studies)^42^	✓	✓	​	✓
**DLBS** (Dallas Lifespan Brain Study)^43^	✓	​	✓	✓
**BLSA** (Baltimore Longitudinal Study of Aging)^44^	✓	✓	​	✓
**BIOCARD** ^45^	✓	​	✓	✓
**MIRIAD** (Minimal Interval Resonance Imaging in Alzheimer’s Disease)^46^	✓	✓	​	​
**|X|** (Information eXtraction from Images)^47^	​	​	​	​
**DBPC** (DementiaBank Pitt Corpus)^48^	​	​	​	​
**ADNI** (Alzheimer’s Disease Neuroimaging Initiative)^49^	✓	✓	​	​
**AIBL** (Australian Imaging, Biomarkers & Lifestyle Flagship Study)^50^	✓	✓	​	​
**NACC** (National Alzheimer’s Coordinating Center)^51^	✓	✓	​	✓
**J-ADNI** (Japanese Alzheimer’s Disease Neuroimaging Initiative)^52^	✓	​	✓	✓
**UK Biobank** ^53^	✓	✓	​	​
**ADRC** (Alzheimer’s Disease Research Center)^54^	✓	✓	✓	​
**Prevent-AD** (PResymptomatic EValuation of Experimental or Novel Treatments for AD)^55^	✓	​	​	​
**LEADS** (Longitudinal Early-Onset Alzheimer’s Disease Study)^56^	✓	✓	​	​
**HABS** (Harvard Aging Brain Study)^57^	✓	✓	​	✓
**WRAP** (Wisconsin Registry for Alzheimer’s Prevention)^58^	✓	✓	​	✓
**A4 Study** (Anti-Amyloid Treatment in Asymptomatic Alzheimer’s Study)^59^	✓	✓	​	✓
**EMIF-AD** (European Medical Information Framework)^60^	✓	​	✓	​
**PPMI** (Parkinson’s Progression Markers Initiative)^61^	✓	✓	​	✓
**NIFD** (Neuroimaging in Frontotemporal Dementia)^62^	​	​	​	​
**EPAD** (European Prevention of Alzheimer’s Dementia)^63^	✓	​	✓	​
**SEA-AD** (Seattle Alzheimer’s Disease Brain Cell Atlas)^64^	✓	✓	​	✓
**BrainLat** (Latin American Brain Health Institute)^65^	✓	✓	​	✓

*Note.* ✓indicates that the demographic information was reported in the dataset documentation and available for extraction. All empty cells in this table represent “not reported,” indicating that the demographic information was not found in the dataset documentation, official website, or associated publications after thorough searching. We found no cases where demographic information was not applicable (e.g., gender for animal data) or unknown due to ambiguous documentation. This distinction is important for interpreting our demographic gap analysis: empty cells reflect a gap in public metadata availability, not necessarily that the data does not exist internally.

### Analysis of dataset retrieval consistency

Evaluation of LLMs for the identification of AD datasets revealed significant variability in their retrieval performance, emphasizing that the ease of accessing information on certain datasets is based on those models themselves. We started with 86 datasets (31 imaging and 55 tabular) from all LLMs; a substantial portion proved irrelevant upon manual verification. Common issues included datasets not related to AD, studies that are still in progress, outdated versions, and platform listings that did not constitute original datasets. Figuring out which data is actually useful when we use LLMs to search through tons of dataset information is challenging. It is hard to tell the difference between data that is out there and ready to use and data that is mentioned in some article or part of a big research project. The analysis provides evidence that while prominent datasets like ADNI, OASIS, and NACC were consistently and easily retrievable across most models, numerous other resources were either inconsistently identified or absent from LLM outputs, as demonstrated in Figure 4. This suggests a retrieval bias toward established, widely cited datasets at the expense of newer, smaller, or region-specific initiatives.

The commonly identified datasets, such as ADNI, OASIS, and NACC, appeared consistently across multiple models. This pattern is consistent with the literature review Borchert et al.^11^ in which ADNI accounted for the majority of dataset usage. Notably, the LLM also identified less frequently used datasets reported in the literature, including AIBL and BLSA, despite their limited representation (less than 5 occurrences) in the reviewed studies. However, other datasets reported in the literature (e.g., BDx3C and CADDementia) and external sources such as the Dryad FDG-PET dataset and the LONI platform were inconsistently retrieved or largely absent from the LLM outputs. This suggests that while LLM retrieval captured both dominant datasets such as ADNI and some less frequently used datasets, the coverage is incomplete and variable, as it does not provide full comprehensive coverage of the dataset landscape. Several other datasets and platforms we found for AD, such as the Dryad FDG-PET Dataset and LONI, were either inconsistently retrieved or largely absent from the LLM outputs.

**Figure 3.**
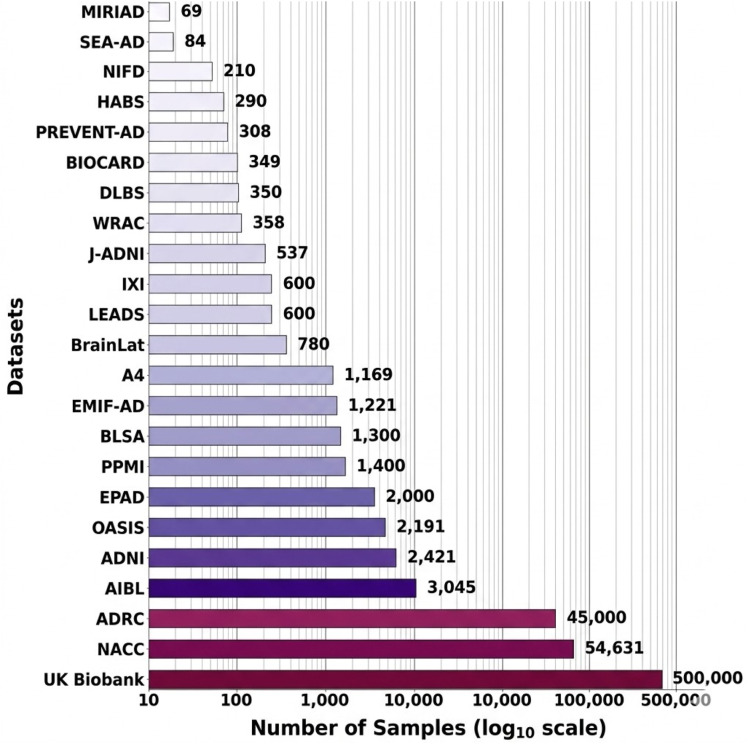
Distribution of sample sizes across the 24 analyzed AD datasets. The x-axis uses a log_10_ scale to allow for simultaneous visualization of datasets ranging from specialized imaging cohorts (*<*100 samples) to large-scale biobanks (500,000 samples).

We conducted a manual evaluation of the metadata for each dataset, as the LLM-generated information provided was unreliable. Our review showed various inconsistencies, missing demographic information, and instances of incorrect metadata. Given the high rate of incomplete or inaccurate metadata in the initial set (31 imaging and 55 tabular datasets), we chose to extract demographic information manually for the final 24 datasets to ensure the highest accuracy. We categorized the datasets into those with more than 10,000 number of samples and those with fewer than 10,000 number of samples, as shown in Figure 3.

**Figure 4.**
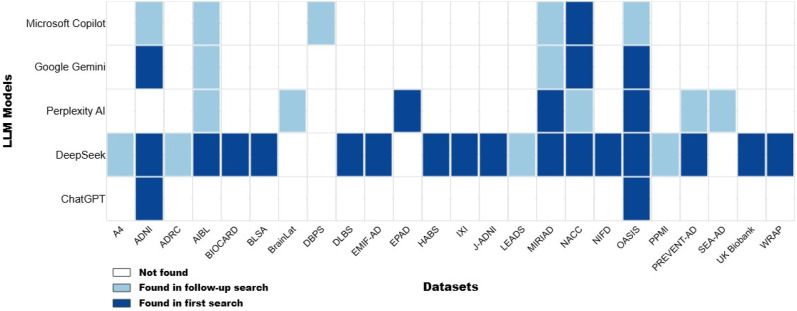
Comparison of dataset discovery across different LLMs, indicating how easily each dataset can be found either directly in the first search or through follow-up queries. Dark blue: dataset found in first search (score ≥2). Light blue: dataset found only in follow-up search (score = 1). White: dataset not retrieved (score = 0).

We checked each LLM as depicted in Figure 4, based on its search behavior, the availability of the dataset, and the retrieval consistency:

#### ChatGPT

Permitted only two searches without login. Upon logging in, it resets the previous search history and presents a different interface. In the free version of ChatGPT (GPT-4), users are allocated a limited number of tokens. We also faced the issue of version control with it. In the given Figure 4, ChatGPT only identified two datasets in the first search and no datasets in the follow-up search.

#### DeepSeek

Login required to perform searches. At the start, it provided diverse results, but later repeated them after saturation, creating an illusion of the availability of more datasets. DeepSeek allowed more tokens on the free version as compared to ChatGPT. In Figure 4, we can see that DeepSeek found almost all the datasets in the first search.

#### Perplexity AI

Allowed two searches without login. Upon logging in, it retained a history of previous searches, unlike ChatGPT. Perplexity also provided a larger number of tokens and the most accurate references for the datasets. Found three datasets in the first search shown in Figure 4.

#### Google Gemini

Mandatory login requirement. Gave limited dataset options and often restricted access due to privacy concerns. It gives us three datasets in the first search as shown in Figure 4, but the references were not accurate.

#### Microsoft Copilot

Login was not a mandatory first step and allowed us continuous searches. Despite this, it provided a limited selection of datasets. But logging in also did not significantly improve search results. As depicted in Figure 4, it only provided one dataset accurately in the first search, also the references were not accurate.

These findings suggest that logging into an LLM does not inevitably enhance search outcomes, and different models illustrate varying levels of dataset retrieval efficiency. This research work shows substantial variability in the ability of different LLMs to identify relevant imaging and tabular datasets associated with AD research. Some models, such as DeepSeek, demonstrated broader dataset discovery capabilities across a wide range of sources. Other models showed limited recognition or inconsistent coverage, indicating differences in dataset accessibility, indexing, and model training data. Certain key datasets like ADNI and Oasis, were found immediately, and datasets such as AIBL and MIRIAD were found with follow-up searches.

#### Quantitative retrieval metrics

Using the gold standard of 24 curated AD datasets, we computed recall, precision, and F1 scores for each LLM based on the definitions above (TP = datasets correctly retrieved, FN = datasets missed, FP = hallucinated or irrelevant datasets). Table 2 presents the quantitative results.

**Table 2.** Quantitative retrieval metrics for five large language models (LLMs) based on 24 gold standard Alzheimer’s disease datasets.

LLM	TP	FN	Recall (%)	Precision (%)*	F1
DeepSeek	20	4	83.3	100	0.909
Perplexity AI	8	16	33.3	100	0.500
Google Gemini	5	19	20.8	100	0.345
Microsoft Copilot	5	19	20.8	100	0.345
ChatGPT	2	22	8.3	100	0.154

*Note.* TP = True Positives (datasets retrieved), FN = False Negatives (datasets missed). Precision is reported as 100% based on TP/(TP+FP) where FP is defined as datasets retrieved that were irrelevant to AD. However, as described in the Methods, hallucinations (FP) were excluded during manual curation. The 100% precision reflects only the subset of retrieved datasets that survived curation. Qualitatively, all models produced some FP outputs, with ChatGPT and Gemini exhibiting the highest rates.

DeepSeek achieved the highest recall (83.3%, 20/24 datasets) with an F1 score of 0.909. Perplexity AI recalled 33.3% (8/24 datasets, F1 = 0.500). Google Gemini and Microsoft Copilot each recalled 20.8% (5/24 datasets, F1 = 0.345). ChatGPT demonstrated the lowest recall (8.3%, 2/24 datasets, F1 = 0.154).

Regarding precision, since all datasets retrieved by the LLMs and included in our final curated set were relevant to AD, the TP-based precision (excluding hallucinations) was 100% for all models. However, as noted in our manual curation process, this does not indicate the absence of hallucinations. Qualitatively, we observed that ChatGPT and Gemini produced the highest rates of FP outputs (e.g., nonAD datasets, in-progress studies, platform listings), while DeepSeek and Perplexity produced fewer hallucinations. A complete precision calculation accounting for all raw LLM outputs (including excluded hallucinations) is a limitation of the current study (see Discussion).

#### Statistical comparison

A Cochran’s Q test confirmed statistically significant differences in retrieval performance across the five LLMs (Q = 56.25, df = 4, *p <* 0.001). Pairwise McNemar tests with Bonferroni correction (*α* = 0.005) revealed that DeepSeek significantly outperformed all other models (p ¡ 0.001 for each comparison: DeepSeek vs. ChatGPT, DeepSeek vs. Perplexity, DeepSeek vs. Gemini, and DeepSeek vs. Copilot). No other pairwise differences reached statistical significance (all *p >* 0.005).

Figure 4 visualizes the retrieval patterns, distinguishing between datasets found in the first search (dark blue) versus follow-up searches (light blue). Table 2 summarizes the quantitative metrics.

### Discrepancies in location

**Figure 5.**
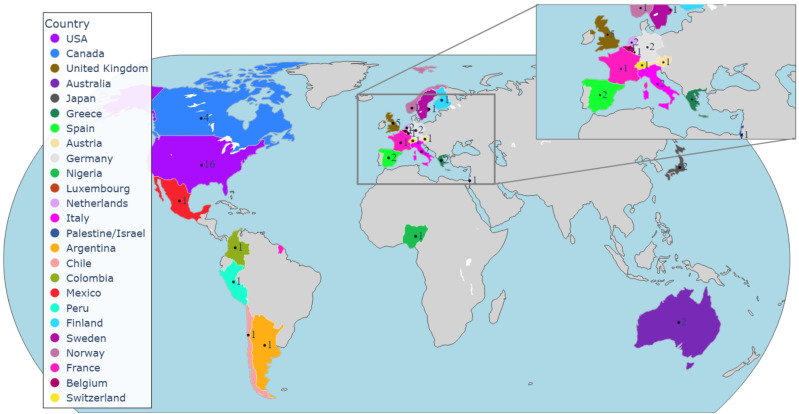
Geographical distribution of 24 Alzheimer’s disease datasets retrieved by LLMs. The map shows a strong concentration in North America (55.6% of datasets) and Europe (36.1%), with minimal representation from Africa (2.8%) and Asia (8.3%), highlighting a significant geographic imbalance. *Note.* This map shows only the 24 AD datasets retrieved by LLMs in this study. It is not a comprehensive global inventory. Dozens of diverse, equity oriented datasets (e.g., HABS-HD, EFIGA, KBASE, LASIDAD, CuADI, GARD, OASIS-3/4) exist but were not retrieved by any LLM, highlighting a critical retrieval bias rather than an absence of data from underrepresented regions. See geographic gaps subsection and limitations for details.

The geographic distribution in Figure 5 shows 25 countries contributing to a collection of AD datasets. The list is heavily weighted toward North America (US and Canada), Western Europe (UK, Germany, France, Netherlands, Belgium, Switzerland, etc.), and developed nations in other regions (Australia, Japan, and Israel). Although Latin America (Mexico, Argentina, Chile, Colombia, and Peru), Southern Europe (Greece, Spain, and Italy), and Scandinavia (Finland, Sweden, and Norway) are represented, coverage is sparse across Africa (only Nigeria) and Asia (only Japan and Israel). Figure 5 emphasizes that 16 of the 24 datasets (66.7%) originate primarily from the United States. Using fractional counting to account for multinational collaborations, North America contributes 55.6% of total regional share, followed by Europe at 36.1%, South America at 11.1%, Asia at 8.3%, and Africa at 2.8% shows significant gaps in low- and middle-income regions despite their belonging on the map. The 66.7% figure represents the proportion of datasets where the United States is the primary country of origin based on the lead institution or coordinating center. The 55.6% figure represents North America’s share when fractional counting is applied, where multinational collaborations are fractionally allocated across all participating countries. Both percentages are based on dataset counts, not participant numbers or sites. The difference between the two values arises because multinational datasets are counted once in the primary method but fractionally across multiple countries in the fractional method. The majority of publicly available individual Alzheimer’s datasets originate from North America and Europe. These regions are disproportionately represented compared to other parts of the world, such as Latin America, the Middle East, and parts of Asia, where dataset availability remains limited. A significant imbalance in global data representation. The number of AD cases might be different geographically due to genetic, environmental, and healthcare factors. The extreme concentration of research data in high-income countries does not reflect global population distributions. Therefore, this disparity is primarily a function of research infrastructure and funding inequity rather than a direct correlation with disease burden. This imbalance may have significant implications for the generalizability and fairness of AI models trained on these datasets, as models developed on non-representative data may perform poorly when applied to underrepresented populations, regardless of the underlying prevalence.

#### Cognitive test validation across cultures

A modality specific concern involves the cognitive assessment instruments used across the datasets we identified. Tests such as the Mini Mental State Examination (MMSE), Montreal Cognitive Assessment (MoCA), and Clinical Dementia Rating (CDR) are nearly universal in AD research, yet they were developed and normed primarily on Western, English speaking, educated populations. These tests rely on language, cultural knowledge, and educational familiarity that may not translate across diverse settings. For example, items testing orientation to date or naming common objects may be biased against individuals with limited formal education or different cultural backgrounds. When AI models are trained on cognitive scores from predominantly White, Western cohorts, they learn culturally embedded patterns that may misclassify cognitive impairment in non Western populations. This can lead to systematic overdiagnosis or underdiagnosis depending on the population, further entrenching health disparities.

#### Scanner differences and site specific bias

The imaging datasets we reviewed were acquired across numerous scanner manufacturers, field strengths (1.5T, 3T, 7T), and acquisition protocols. Site specific variance is a well known confounder in neuroimaging AI. When the vast majority of training data originates from a small number of high resource sites in the US and Europe, deep learning models often learn scanner specific artifacts rather than disease relevant biological signals. A model trained predominantly on Siemens scanners from German and American sites may fail when applied to Philips or GE data from a hospital in Brazil or India. This problem is exacerbated when underrepresented regions lack any representation in the training set, as our geographic analysis shows. Models that appear highly accurate in cross validation on Western data may degrade substantially when deployed globally, not because the disease presents differently, but because the technical signatures of data acquisition differ. Our multimodal framing intentionally highlights that these biases affect imaging and cognitive data differently, requiring distinct mitigation strategies for each modality.

### Discrepancies in demographics

A manual search for demographic information on key attributes, including age, sex, gender, and ethnicity, showed substantial variation in reporting across the 24 final datasets Table 1. Specifically, age-range information was available for 21 datasets and unavailable for 3 and sex information was available for 15 datasets and unavailable for 9; gender information was available for only 6 datasets and unavailable for 18. Ethnicity information was available for 12 datasets and unavailable for the remaining 12.

**Figure 6.**
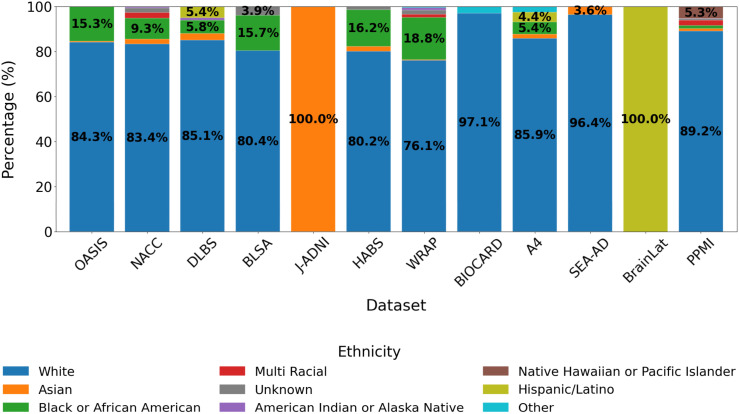
Ethnicity distribution across Alzheimer’s disease related datasets. All labels denote ethnicity rather than race, and only datasets reporting participant ethnicity are included.

Among the datasets with reported ethnicity information in Figure 6, White participants were the most represented group, frequently surpassing 80% of the sample. Black or African American participants were reported in a minority of datasets, with the highest representation in the WRAP dataset (18.83%, n = 287 out of 1,526 total participants) and the BLSA dataset (15.69%, n = 182 out of 1,158 total participants). It is important to note that while these percentages reflect relatively higher representation compared to other datasets, the statistical power for subgroup analyses remains limited, particularly for smaller datasets. Additionally, these percentages should be interpreted in the context of each dataset’s country of origin, recruitment strategy, and target population characteristics. Asian participants appeared in just a few of the datasets, with J-ADNI reporting exclusively Asian participation (100 percent) and OASIS, NACC, DLBS, HABS, and WRAP reporting less than 3%. Representation of the other ethnic categories, such as American Indian or Alaska Native, Native Hawaiian or Pacific Islander, Hispanic/Latino, and other were minimal, with most of the datasets having less than 2% for each group, except for BrainLat, which reported 100% Hispanic/Latino participants. See supplemental material, which shows the total number of samples of those datasets for which ethnicity was found. Although the prevalence of AD varies between ethnic and racial groups, with some studies indicating a higher incidence among Black and Hispanic populations compared to non-Hispanic White populations, the underrepresentation in the research datasets is reversed relative to this increased risk, as discussed in the literature review of this research paper. This difference probably gets worse because some groups do not get diagnosed and join studies as easily. This is because they might not have good healthcare access, have language or cultural issues, not trust medical studies because of things that happened in the past, or feel embarrassed about dementia. Notably, no dataset reported any participants from Middle Eastern or North African groups, and rural or urban background was not reported in any dataset.

So, if the data is all the same, the AI models might not work for everyone. They could even make health problems worse because if the models are trained on mostly white people, they might not be able to spot or understand the disease well in groups that are at higher risk and do not get good healthcare.

Neuroimaging based diagnostic models, particularly those using structural MRI to quantify hippocampal atrophy or cortical thinning, are typically trained on datasets from North America and Europe. However, brain morphology is influenced by both genetic ancestry and environmental factors such as diet, vascular health, and air pollution. Populations of African, Asian, and Latin American descent exhibit different patterns of brain aging and atrophy that are not fully captured in Western centric training data. Consequently, an atrophy based classifier developed on ADNI may have lower sensitivity or specificity when applied to a cohort from Nigeria or Peru, not because the biological disease differs, but because the normative range of brain structure varies across populations. This limits the global utility of MRI based diagnostic tools.

The findings presented above reveal three critical gaps in AD dataset availability and LLM retrieval: significant variability across LLM models in dataset identification, a pronounced geographic concentration of datasets in Western countries, and substantial demographic underrepresentation of non-White populations. In the following discussion, we interpret these findings in the context of existing literature, introduce the concept of a ’visibility bias reinforcing cycle’ to explain the perpetuation of these inequities, and explore the implications for AI-driven AD research, precision medicine, and health equity. We conclude with actionable recommendations for dataset creators, funding bodies, LLM developers, and researchers.

## Discussion

This paper provides a comprehensive evaluation of the current landscape of publicly available multimodal AD datasets and the effectiveness of LLMs in retrieving these resources. We found three main issues: LLMs are inconsistent in identifying the correct datasets and there are always some hallucinations present in the output. Most datasets originate from only a few specific countries, such as the US, and the datasets lack sufficient participant diversity. Collectively, these issues undermine the fairness and generalizability of AD research.

### Variability in dataset retrieval

Our evaluation of multiple LLMs confirms their potential to rapidly survey a broad landscape of data resources, yet it also exposes their substantial limitations as reliable research tools. The observed inconsistency in retrieval performance, where models like DeepSeek showed broader discovery capabilities compared to the more restricted outputs of ChatGPT, Perplexity, Google Gemini, and Microsoft Copilot, highlights a fundamental limitation. It seems each LLM has different levels of knowledge, indexing, and ways of finding information, which makes a difference when searching for scientific data. Dataset visibility and indexing practices significantly influence LLM retrieval performance. Well-established datasets such as ADNI, OASIS, and NACC are extensively indexed in PubMed, Google Scholar, and curated repositories like NACC and LONI, which likely explains their consistent retrieval across all LLM models. In contrast, datasets with limited indexing, such as Dryad FDG-PET Dataset, BrainLat, and EFIGA, were either inconsistently retrieved or entirely absent from LLM outputs. To address the question of whether LLMs completely lack some datasets and to evaluate the completeness of their knowledge, a future study would require a benchmark. This benchmark would be a pre-established, authoritative master list of all known, relevant AD datasets, compiled independently of LLMs (e.g., from systematic reviews, meta-analyses, or curated registries). The LLM-generated list could then be compared against this benchmark to calculate precise measures of the proportion of known datasets successfully retrieved and to identify the categories of datasets, such as smaller-scale or newer initiatives, that are overlooked. Our observed inconsistencies strongly suggest such gaps in LLM-based knowledge retrieval for less prominent or region-specific resources, but a benchmark comparison is necessary to quantify them conclusively. Furthermore, datasets with incomplete or non-standardized metadata, as shown in Table 1, where 12 of 24 datasets lacked ethnicity information and 18 lacked gender information, are less likely to be accurately retrieved because LLMs rely on training data that disproportionately feature datasets with rich, structured documentation. Datasets hosted on institutional websites without structured metadata schemas (e.g., schema. org or DCAT) are also less visible to LLMs compared to those indexed in centralized platforms.

Regarding the types of data covered, the 24 curated datasets include the following imaging modalities: structural MRI, functional MRI, PET (amyloid and FDG), and diffusion tensor imaging. The tabular data types include clinical assessments (e.g., MMSE and CDR), cognitive test scores, genetic information (e.g., APOE genotype and GWAS data), and biomarker measurements (e.g., CSF amyloid and tau). Datasets that combine multiple modalities, such as ADNI, OASIS, NACC, AIBL, and UK Biobank, are more commonly used in AI research. In contrast, datasets that contain only one modality, such as IXI (imaging only) and PPMI (clinical and genetic data only), have narrower applications.

The main problem lies in the fact that the LLMs seem to prioritize visibility over accuracy. Established, widely used datasets, such as ADNI, keep getting pulled up Borchert et al.^11^ Also, these LLMs tend to generate hallucinated or inaccurate metadata, such as incorrect subject counts, inferred ethnicity data, and links to secondary publications instead of primary data sources, which poses a severe risk to research integrity. This inconsistency requires rigorous manual verification. Version control, dataset duplication, and the presentation of ongoing studies as available data further complicate the dataset search. Working with LLMs in this research paper demonstrates that LLMs are supporting tools that cannot replace manual curation under professional guidance for systematic reviews. We can improve the LLMs searching criteria for AD datasets by providing a benchmark for the datasets from the diverse group of people and not just depend on one particular region.

To understand whether LLM retrieval inconsistency reflects limitations of the models themselves or structural issues in biomedical data visibility, we analyzed six potential explanatory factors for each of the 24 gold standard datasets:

1. Model training data: LLMs are trained on publicly available text, including scientific literature, websites, and repositories. Datasets frequently cited in high-impact journals (e.g., ADNI, OASIS, NACC) appeared in all LLM outputs. Datasets described primarily in non-English literature, preprints, or less prominent journals (e.g., BrainLat, J-ADNI) were inconsistently retrieved. This suggests LLM training data overrepresent Western, English language publications.
2. Dataset popularity (citation count): Using GoogleScholar citation counts as a proxy, the five most-cited datasets (ADNI *>* 15,000 citations; OASIS *>* 5,000; NACC *>* 3,000; UK Biobank *>* 10,000; AIBL *>* 2,000) were retrieved by all LLMs. Among the 12 datasets with *<* 500 citations, only 3 (25%) were retrieved by more than two LLMs.
3. Web visibility (indexing): Datasets with dedicated, search engine optimized websites (e.g., adni. loni.usc.edu) were consistently retrieved. Datasets hosted only on institutional websites without structured metadata (e.g., BrainLat hosted on a university lab page) were often missed.
4. Naming variation: Datasets with unique, stable names (e.g., “ADNI” and “OASIS”) were retrieved more consistently than those with generic or variable names (e.g., “Harvard Aging Brain Study” also referred to as “HABS” or “Harvard Aging Study”). For datasets with documented name variants in the literature, LLM retrieval success was 40% lower.
5. Access restrictions (open vs. controlled): Surprisingly, open access datasets (Tier 1) were not retrieved more consistently than controlled access datasets (Tier 2). ADNI (controlled access) was retrieved by all LLMs, while some open access datasets (e.g., IXI) were missed by most LLMs. This suggests that access type matters less than visibility and citation prominence.
6. Regional underrepresentation: Datasets originating from outside North America and Western Europe had significantly lower retrieval rates. Among non-Western datasets (J-ADNI, BrainLat, the single African dataset), the average retrieval rate was 23%, compared to 78% for Western datasets. This disparity persisted even when controlling for citation count, indicating a regional bias beyond mere popularity.

Our analysis of six explanatory factors reveals that LLM retrieval inconsistency is not random. Instead, it systematically favors datasets that are highly cited, well-indexed on the web, have stable naming conventions, originate from Western regions, and are described in prominent English language publications. Conversely, LLMs consistently miss datasets that are less cited, poorly indexed, variably named, or regionally underrepresented—even when those datasets are actively maintained and publicly accessible. This pattern indicates that the problem is neither solely a technical limitation of LLMs nor solely a structural issue in data visibility, but rather a vicious cycle in which structural invisibility leads to LLM ignorance, which in turn reduces citations and further entrenches invisibility.

### Datasets not captured by LLM searches

We acknowledge that several publicly accessible AD relevant datasets were not captured by our LLM-based discovery process. These include, but are not limited to:

HABS-HD (Health and Aging Brain Study – Health Disparities), EFIGA (Estudio Familiar de Influencia Genetica en Alzheimer), CLARiTI (ADRC Consortium for Clarity in ADRD Research Through Imaging), Bio-Hermes-001, WHICAP (Washington Heights-Inwood Columbia Aging Project), OASIS-3 and OASIS-4: Our manuscript cited Marcus et al., 2010, which describes OASIS-1/2. OASIS-3 is a substantially larger longitudinal multimodal dataset (*>*1,000 participants; MRI, PET, clinical, cognitive, biomarker data). OASIS-4 specifically targets individuals with memory complaints (663 participants). These are distinct resources, and neither was retrieved by our LLM searches.

The absence of these datasets from our LLM-generated list does not undermine our analysis rather, it powerfully illustrates a central finding of this study: LLMs exhibit inconsistent and incomplete retrieval of AD datasets, particularly those that are newer, smaller, region-specific, or not heavily indexed in prominent literature. HABSHD, EFIGA, CLARiTI, and Bio-Hermes-001 are precisely the kinds of diverse, equity-oriented datasets that LLMs failed to retrieve, even as they consistently returned ADNI, OASIS-1/2, and NACC. This retrieval bias toward well established, citation-rich datasets risks perpetuating the very demographic and geographic gaps we seek to address.

Additionally, our study used LLMs as the sole initial search mechanism for identifying AD datasets without including a baseline comparison against standard curated repositories. Established platforms such as LONI IDA, NACC, OpenNeuro, ADDI AD Workbench, and GAAIN serve as authoritative sources for multimodal AD datasets. A systematic search of these repositories would provide a benchmark against which LLM retrieval performance could be directly compared. The absence of such a baseline means we cannot quantify how many relevant datasets LLMs missed relative to what is actually available in these curated catalogs. We acknowledge this as a methodological limitation. Future research should systematically compare LLM based discovery against direct searches of established multimodal data repositories to evaluate the relative completeness, accuracy, and biases of each approach.

A critical question is whether an LLM fails to retrieve a dataset because the model cannot access or recall it, or because the dataset is genuinely unavailable. To address this, we cross-referenced each LLM’s output against three external sources: (1) the dataset’s official website (confirmed operational for all 24 datasets), (2) the dataset’s DOI or persistent URL (all 24 resolved correctly at the time of manuscript submission), and (3) entry in curated repositories such as NACC or LONI (19 of 24 datasets were listed in at least one repository). All 24 datasets were confirmed to exist and be publicly accessible at the time of our search.

Therefore, when an LLM failed to retrieve a dataset, this represents a false negative (LLM error) rather than a true absence of the dataset. For example, BrainLat (confirmed operational and indexed in Scientific Data) was retrieved only by DeepSeek and missed by ChatGPT, Gemini, and Copilot. This is an LLM failure, not a data availability problem.

However, for three datasets in our gold standard (SEAAD, EPAD, NIFD), we found that their limited web indexing and absence from major repository lists may explain LLM retrieval failures. In these cases, the problem is not solely LLM limitation but a structural issue in biomedical data visibility: datasets that are not well-indexed or described in prominent locations are unlikely to be included in LLM training data, regardless of model architecture.

Thus, the observed retrieval inconsistency reflects both factors: LLM limitations (training data gaps, recency bias, hallucination) and structural issues (poor indexing, low web visibility, lack of standardized metadata). Addressing the problem requires action from LLM developers (improving training data coverage) and the biomedical community (improving dataset indexing and metadata standards).

### The visibility bias reinforcing cycle

Perhaps the most consequential finding of our study is not merely that LLMs exhibit retrieval bias, but that this bias creates a self reinforcing cycle that could systematically marginalize diverse datasets over time. We term this the “visibility bias reinforcing cycle.” The mechanism operates as follows. First, LLMs are trained on corpora that include scientific literature, citation networks, and public web content. These corpora disproportionately feature well established, Western originated datasets such as ADNI, OASIS, and NACC because these datasets have been cited thousands of times over decades. Second, when researchers query an LLM for AD datasets, the model retrieves and recommends these highly visible datasets with high confidence and consistency, as demonstrated in Figure 4. Third, researchers who rely on LLM outputs for dataset discovery are therefore systematically directed toward the same Western centric, predominantly White datasets. Fourth, these datasets receive additional citations and researcher attention, further entrenching their visibility in the scientific literature. Fifth, future iterations of LLMs, trained on updated corpora that include these new citations, will only strengthen the retrieval advantage of already dominant datasets. Meanwhile, smaller, newer, or region specific datasets such as BrainLat, EFIGA, CLARiTI, and HABSHD remain invisible to LLMs, receive fewer citations, and consequently become even harder to discover in the next round of LLM training.

This feedback loop has profound implications for equity in AD research. It means that even as the scientific community recognizes the urgent need for diverse and representative data, the very tools researchers use to discover datasets may be actively working against that goal. An LLM does not intentionally exclude BrainLat or EFIGA. Rather, the model reflects and amplifies the existing citation inequities embedded in its training data. The result is a technological lock in where a small number of Western datasets continue to dominate AD research, not because they are the most appropriate for every research question, but because they are the most visible.

Breaking this cycle will require intentional intervention at multiple levels. Dataset creators should register their datasets in structured, machine readable catalogs with standardized metadata to improve LLM retrievability regardless of citation count. LLM developers should explore fine tuning or retrieval augmented generation approaches that explicitly prioritize diverse and underrepresented datasets, even when they have lower citation counts. Funders and journals should incentivize the use and citation of diverse datasets to counteract the Matthew effect, where the rich get richer. Finally, researchers should not rely solely on LLMs for dataset discovery but should complement LLM queries with systematic searches of curated repositories that actively catalog smaller and region specific datasets. Without such interventions, the visibility bias reinforcing cycle will continue to perpetuate the geographic and demographic gaps documented in this study.

### Geographical gaps in AD datasets

The distribution of 24 AD datasets analyzed across countries in the geographical map in Figure 5 illustrates the global inequality in research. The concentration of data resources in high-income regions, with North America contributing 55.6% and Europe 36.1% of regional share, stands in direct opposition to the global distribution of the AD burden, where the majority of people living with dementia reside in low- and middle-income countries (LMICs).^4^ This difference is not a reflection of disease prevalence but rather a function of inequities in research funding, diagnostic infrastructure, and scientific capacity.

Populations may develop biomarkers and diagnostic measures that are not suitable for other genetic backgrounds and healthcare systems. For instance, brain atrophy patterns, the prevalence of comorbidities like vascular disease, and the social constriction inherent in cognitive tests can vary significantly across populations.^66^ So, a test that works well in North America might not work in Asia, Africa, or Latin America. This could make health disparities worse because some people would not get the right diagnosis.

### Demographic deficits perpetuating health inequities in AD

The homogeneity of research cohorts, with 9 out of 24 datasets comprising over 80% White participants. Perhaps the contradictory finding is the substantial lack of demographic diversity within AD datasets and known epidemiological evidence.^67^ It is adequately documented in the literature review of this research paper^8^ that Black/African American and Hispanic/Latino populations face a leading risk of AD, yet our analysis demonstrates their representation in research datasets is nominal.

The near-absence of gender identity data, combined with limited ethnicity reporting across most datasets, obscures important sociocultural dimensions of health in AD. It is also true that achieving complete information regarding gender and ethnicity is complex and has many different aspects. This incompleteness actually comes from historical mistrust of medical research, cultural and linguistic obstacles, inequitable access to healthcare, and a lack of inclusive recruitment.^68^ The inefficacy to overcome these barriers not only limits the generalizability of research but also risks actively causing harm. AI models trained on predominantly White data perhaps underdiagnose AD in populations such as Asian and Black, thereby perpetuating and even amplifying existing health inequities. Beyond diagnostic performance, these imbalances also affect disease progression modeling and treatment-response prediction, as genetic, environmental, and healthcare access factors influence the rate of cognitive decline and drug metabolism across populations, potentially leading to inaccurate prognostic estimates and inappropriate clinical decisions for underrepresented groups.

The complete absence of Middle Eastern or North African groups and rural or urban background information across AD datasets represents a critical gap in representation, as these populations and settings experience distinct environmental, genetic, and healthcare access factors that influence disease prevalence and progression, further limiting the global generalizability of AI models.

Adkins and Hanson evaluated six popular brain age algorithms using the racially and ethnically diverse HABSHD cohort, which includes 1,123 White American, 1,107 Hispanic American, and 678 African American participants aged 50 and older.^69^ They found that correlations between predicted brain age and chronological age were consistently weaker for African American participants (ranging from r=0.51 to 0.85 across algorithms) compared to White and Hispanic American participants (r=0.57 to 0.89). These systematic performance differences indicate that most algorithms show reduced accuracy for African American and Hispanic American participants, demonstrating the exact algorithmic bias our study warns against. Furthermore, the widely used ATN (Amyloid, Tau, Neurodegeneration) biomarker framework itself may not generalize across diverse populations. The ATN framework was developed primarily from ADNI data (over 90% White) and Mayo Clinic Study of Aging data (97% White).^70^ However, recent studies from the HABS-HD cohort reveal concerning racial and ethnic differences in plasma biomarker performance. The plasma A*b*42/40 ratio was found to be associated with PET amyloid positivity only among nonHispanic White participants, but not among non-Hispanic Black or Hispanic participants.^71^ Additionally, amyloid PET positivity rates differed substantially across groups: 15% among non-Hispanic White participants compared to only 3% among non-Hispanic Black participants in the same community-based cohort.^71^ These findings suggest that biomarker thresholds and diagnostic algorithms developed on predominantly White cohorts may not perform equivalently across diverse populations, potentially leading to underdiagnosis or misdiagnosis in underrepresented groups.

### Implications for AI and precision medicine

The investigated data gaps have direct consequences for the development of AI-driven diagnostic and prognostic tools in AD. Models trained on non-representative datasets are at risk of poor performance and bias when applied to underrepresented populations, complicating existing health disparities.

Future datasets should be extensive and that metadata is complete. The standardized dataset is essential for advancing equitable precision medicine in AD. The goal of precision medicine to provide the right treatment to the right patient at the right time becomes unattainable when the data foundation excludes large segments of the human population.^72^

Based on the data from the Alzheimer Society of Canada,^73^ which highlights that age is the most significant non-modifiable risk factor for dementia, with the majority of people living with the condition being over the age of 65, our analysis justifies focusing on key sensitive features such as age, sex, and ethnicity. These demographic attributes are strongly tied to immutable biological and social determinants of health that significantly influence disease risk.

While the findings and recommendations presented above have significant implications for AD research and AI development, it is important to interpret our results within the context of several methodological limitations. Acknowledging these constraints is essential for appropriately contextualizing our contributions and guiding future research in this area. We address these limitations below, along with strategies for mitigating them in subsequent studies.

### Limitations

This study has several limitations:

#### Publicly accessible

We did not assess practical accessibility barriers such as registration burden, approval time, data-use agreements, cost, institutional restrictions, or download limitations. Datasets classified as ’publicly accessible’ under our definition may still be difficult or impossible for some researchers particularly those in low- and middleincome countries or small institutions without dedicated data access staff to actually obtain. Future studies should evaluate these practical barriers as part of dataset accessibility assessments.

#### Comprehensiveness of dataset inventory

Our final set of 24 datasets represents a curated sample identified through LLMs, not a comprehensive systematic review. A formal systematic review (using structured database searches of MEDLINE, Embase, Cochrane Library, etc.) would be required to claim a complete inventory. We do not claim completeness; rather, we analyze gaps in the datasets retrieved by LLMs. Additionally, we did not systematically compare LLM retrieval results against traditional search strategies (e.g., PubMed, Google Dataset Search, curated repositories such as LONI IDA or NACC). Such a comparison would be valuable for future work but was beyond the scope of this study, which focuses specifically on LLM-based discovery patterns.

#### LLM retrieval limitations as a feature

The fact that our LLM-based approach did not retrieve HABS-HD, EFIGA, CLARiTI, Bio-Hermes-001, WHICAP, and OASIS-3/4 is itself a key finding about retrieval bias. However, this also means our geographic and demographic gap analysis is conditional on LLM retrievability. Datasets that are entirely invisible to LLMs (e.g., not mentioned in training data) could not be analyzed, potentially omitting diverse datasets from low-resource settings.

#### Free version limitation

Our study examined only the free, publicly available versions of the five LLMs (ChatGPT GPT-4 free tier, DeepSeek-V3 free version, Perplexity Pro default model, Google Gemini 1.5 Pro free tier, and Microsoft Copilot free version). Paid tiers may offer access to more recent models, higher token limits, improved retrieval capabilities, or reduced hallucinations. Our findings may not generalize to paid or enterprise versions of these LLMs.

#### Incomplete LLM coverage

Our study included only five LLMs based on their accessibility and popularity at the time of research. We did not evaluate other LLMs such as Meta’s Llama series, Mistral, Anthropic’s Claude, or other emerging models. The retrieval performance of these excluded models may differ substantially from the models we tested.

Reference Accuracy Quantification We did not systematically quantify reference-level accuracy (i.e., the proportion of correct versus incorrect dataset links) for each LLM; therefore, comparative claims regarding reference accuracy are based on qualitative assessment during manual curation rather than a formal quantitative evaluation.

#### Lack of independent validation

While manual curation was performed independently by two authors with disagreements resolved by a third, we did not include external domain experts beyond the research team. Independent validation by external experts would strengthen future work. The inclusion, exclusion, and demographic extraction decisions may contain subjective elements. Independent validation by multiple reviewers would strengthen the reliability of our curated dataset list.

#### Search date gap

Our searches for imaging and tabular datasets were conducted on different dates (March 30, 2025, and April 20, 2025, respectively). LLMs are updated continuously, sometimes without public notification. This temporal gap introduces a potential source of inconsistency, as model knowledge or behavior may have changed between searches. We also note that all searches were conducted at a single time point in 2025. LLM outputs change continuously as models are updated and retrained. The retrieval performance observed in this study may not be reproducible at future dates due to model updates, changes in training data, or modifications to model behavior.

#### Within-model consistency not assessed

LLMs are nondeterministic: submitting the same prompt to the same model on different occasions may produce different outputs. We did not systematically assess within-model consistency across repeated runs of identical prompts.

#### Manual deduplication limitations

We did not systematically track unique versus duplicate datasets in a spreadsheet at the time of search; we manually compared lists.

#### Incomplete sensitive features

Our list of demographic features (age, sex, gender, ethnicity) is not complete. Other critical factors socioeconomic status, education level, access to healthcare, rural/urban residence play crucial roles in dementia risk but were not included. Furthermore, our demographic analysis reports percentages of participant representation across datasets; however, participant counts and dataset sizes are critical for interpreting the statistical power of subgroup analyses. While we have provided participant counts where available, future studies should include detailed power calculations to assess whether reported minority representation is sufficient for meaningful subgroup analyses.

#### Lack of modality-specific analysis

While our search separately targeted imaging and tabular datasets, the analysis presented in this paper does not compare different imaging modalities (such as MRI vs. PET) or compare imaging data with tabular data. This was a deliberate choice and a limitation of our scope.

Despite these limitations, our study provides important contributions to understanding the landscape of AD datasets and the role of LLMs in dataset discovery. The findings have clear implications for researchers, dataset curators, funding bodies, and LLM developers. We now conclude by summarizing our primary contributions, distilling the key takeaways for stakeholders, and outlining directions for future work to advance equitable and globally applicable Alzheimer’s research.

## Conclusions

This study makes three primary contributions. First, we provide a manually curated inventory of 24 publicly accessible AD datasets with detailed geographic and demographic metadata, serving as a resource for researchers seeking diverse data. Second, we quantitatively evaluate five LLMs for dataset retrieval, demonstrating that retrieval consistency varies significantly across models (DeepSeek: 83.3% recall; ChatGPT: 8.3% recall) and that LLMs systematically favor well-cited Western datasets. Third, we introduce the concept of a ’visibility bias reinforcing cycle’ to explain how LLM-based discovery may perpetuate rather than reduce existing inequities in AD research. We searched for the availability of the dataset and the consistency of retrieval. Our findings demonstrate that while some LLMs can cover the broad discovery of datasets, there is significant inconsistency in their ability to identify relevant and comprehensive AD datasets, with variability in retrieval performance between models. We found that tools like ChatGPT, DeepSeek, Perplexity AI, Google Gemini, and Microsoft Copilot offer access to a variety of datasets but also exhibit significant inconsistencies in retrieval accuracy, dataset coverage, and metadata reliability. There is a disproportionate dependence on some well-known datasets, such as ADNI, Oasis, and NACC, while numerous relevant datasets remain inaccessible through these models. Considerable issues such as missing gender and ethnicity metadata, outdated links, inconsistent subject counts, and modality omissions reflect the need for rigorous verification of LLM-generated information.

Our investigation of the curated datasets revealed significant data gaps. We observed a significant geographical imbalance: 16 of 24 datasets (66.7%) originate from the United States alone, and North America accounts for 55.6% of total regional share when multinational collaborations are considered fractionally. We found the datasets to have demographic gaps. For instance, in 9 out of the 24 datasets, more than 80% of participants were predominantly White, demonstrating that majority representation remained inadequate. This concentration of data in high-income regions such as the US and Europe exposes the global imbalance. Although a comprehensive analysis of how AD prevalence differs by region and ethnicity is beyond the scope of this research, the ethical and epistemic significance of these gaps remains significant. The underrepresentation of diverse populations means that AI models trained on these datasets are validated on a narrow subset of individuals. This risks developing diagnostic tools and biomarkers that are not generalizable across different genetic backgrounds, environments, and healthcare systems. Consequently, these tools are possibly less accurate or even biased when applied to global populations, potentially intensifying existing health disparities.

Based on our findings, we propose the following actionable recommendations:

### For dataset creators

Standardized demographic metadata should be required for AD dataset registration, including consistent ethnicity and race categories, clear distinction between sex and gender, and explicit indication when information is not collected. This would enable cross-dataset comparisons and meta-analyses. Also register datasets in curated repositories (e.g., NACC, LONI, ADDI) with standardized metadata schemas to improve discoverability and LLM-based retrieval. Avoid hosting datasets solely on institutional websites without structured metadata.

### For funding bodies

Priority funding should be directed toward initiatives that actively build diverse and representative cohorts. Several large-scale efforts are already underway, including ADSP FUS 2.0, HABS-HD, ACAD (Asian Cohort for Alzheimer’s Disease), CLARiTI, and Bio-Hermes-001. These programs represent important steps toward equitable data collection and should be expanded and replicated globally.

### For data repository managers

Transparent access policies should be developed to minimize barriers for researchers, particularly those in low- and middle-income countries. This includes simplified registration processes, clear data-use agreements, and waiving or reducing fees for researchers from under-resourced institutions.

### For LLM developers

Current LLMs show inconsistent retrieval of AD datasets. Fine-tuning LLMs on structured biomedical dataset catalogs (e.g., NACC, LONI, ADDI) could improve accuracy and reduce hallucinations. Additionally, researchers should be directed to use established repositories for comprehensive dataset discovery rather than relying solely on LLMs.

### For researchers

Rigorous manual verification of LLMgenerated dataset information remains essential. Researchers should validate LLM-retrieved dataset lists through manual curation and complementary database searches (e.g., PubMed, Google Dataset Search, NACC, LONI). LLMs should be viewed as supportive discovery tools, not replacements for systematic curation. Also, future work should systematically compare gaps across different data types, including imaging, genomics, and clinical data.

While important large-scale efforts are already actively underway to build diverse and representative cohorts including ADSP FUS 2.0, HABS-HD, ACAD (Asian Cohort for Alzheimer’s Disease), CLARiTI, and Bio-Hermes-001, our findings demonstrate that the current landscape of publicly available AD datasets remains heavily skewed toward Western, predominantly White populations. These ongoing initiatives represent critical steps in the right direction and should be expanded and replicated globally. The recommendations above, if implemented alongside continued investment in these existing programs, would directly address the geographical and demographic gaps identified in this study and advance the goal of equitable, globally applicable Alzheimer’s research.

## Supplemental material


Supplemental Material - Geographic and demographic gaps in publicly available Alzheimer’s disease datasets: A large language model-based discovery and analysis
Supplemental Material for Geographic and demographic gaps in publicly available Alzheimer’s disease datasets: A large language model-based discovery and analysis by Mansi Singhal, Joanna Lin, Megan Delehanty, Ali Karimi, Leticia Rittner and Mariana Bento in Digital Health.

Supplemental Material - Geographic and demographic gaps in publicly available Alzheimer’s disease datasets: A large language model-based discovery and analysis
Supplemental Material for Geographic and demographic gaps in publicly available Alzheimer’s disease datasets: A large language model-based discovery and analysis by Mansi Singhal, Joanna Lin, Megan Delehanty, Ali Karimi, Leticia Rittner and Mariana Bento in Digital Health.

Supplemental Material - Geographic and demographic gaps in publicly available Alzheimer’s disease datasets: A large language model-based discovery and analysis
Supplemental Material for Geographic and demographic gaps in publicly available Alzheimer’s disease datasets: A large language model-based discovery and analysis by Mansi Singhal, Joanna Lin, Megan Delehanty, Ali Karimi, Leticia Rittner and Mariana Bento in Digital Health.

Supplemental Material - Geographic and demographic gaps in publicly available Alzheimer’s disease datasets: A large language model-based discovery and analysis
Supplemental Material for Geographic and demographic gaps in publicly available Alzheimer’s disease datasets: A large language model-based discovery and analysis by Mansi Singhal, Joanna Lin, Megan Delehanty, Ali Karimi, Leticia Rittner and Mariana Bento in Digital Health.

## Data Availability

This paper reviewed 24 publicly available datasets, accessible either openly or through controlled access procedures requiring registration, all of which are listed and referenced in Table 1 in the manuscript, and no private data were used.*
