# Supplementary material for: Geographic and demographic gaps in publicly available Alzheimer’s disease datasets: A large language model-based discovery and analysis
Source: Digit Health. 2026 Jul 21;12:20552076261470698. doi: 10.1177/20552076261470698 (PMC13389145; doi:10.1177/20552076261470698)
Supplement: Supplemental Material - Geographic and demographic gaps in publicly available Alzheimer’s disease datasets: A large language model-based discovery and analysis [file sj-pdf-1-dhj-10.1177_20552076261470698.pdf]

Supplementary Material

This supplementary document provides detailed information about the large language models (LLMs) and the exact prompts used in this study to ensure reproducibility.

S1. LLM Version Information

The following five LLMs were used for dataset discovery:

Table 1. LLM versions used in this study.

LLM	Version
ChatGPT	GPT-4 (free tier)
DeepSeek	DeepSeek-V3
Perplexity AI	Perplexity Pro (default model)
Microsoft Copilot	Copilot, balanced creativity mode (default model)
Google Gemini	Gemini 1.5 Pro (free tier)

S2. Exact Prompts for Imaging Dataset Searches

Search Date: March 30, 2025

Prompt 1:

> *Provide me with a table containing all the imaging datasets related to Alzheimer's disease/dementia. Include details about each dataset, such as a description of the dataset, number of subjects, imaging modality details, preprocessing, age range, sex, gender, ethnicity, location, open access status, whether it is paid or free, linkage datasets consortium or a larger initiative, and the dataset link.*

Prompt 2:

> *Give me all the datasets related to Alzheimer's disease/dementia imaging data as much as you can.*

Prompt 3:

> *Provide me with all available datasets related to Alzheimer's disease/dementia imaging data from around the world.*

S3. Exact Prompts for Tabular Dataset Searches

Search Date: April 20, 2025 Prompt

1:

> *Provide me with a table containing all the datasets related to Alzheimer's disease/dementia tabular datatype. Include details about each dataset, such as a description of the dataset, number of subjects, data types, preprocessing, age range, sex, gender, ethnicity, location, open access status, whether it is paid or free, and dataset link.*

Prompt 2:

> *Give me all the datasets related to Alzheimer's disease/dementia tabular data as much as you can.*

Prompt 3:

> *Provide me with all available datasets related to Alzheimer's disease/dementia tabular data from around the world.*
