# Supplementary material for: Geographic and demographic gaps in publicly available Alzheimer’s disease datasets: A large language model-based discovery and analysis
Source: Digit Health. 2026 Jul 21;12:20552076261470698. doi: 10.1177/20552076261470698 (PMC13389145; doi:10.1177/20552076261470698)
Supplement: Supplemental Material - Geographic and demographic gaps in publicly available Alzheimer’s disease datasets: A large language model-based discovery and analysis [file sj-pdf-2-dhj-10.1177_20552076261470698.pdf]

Table 1: Supplementary Table S1: Comprehensive Inventory of Alzheimer's/Dementia Datasets Identified via LLM-Based Search (March–April 2025).

Dataset	N	Imaging Modalities	Prep	Age	Sex	Ethnicity	Location	Open Access	$	Access Type	LLMs
**ADNI**	2.5K	T1, fMRI, DTI, PET	Y	55-90	Y	Diverse (mostly White)	USA, Canada	Res.	Free	App.	All ex. Perp
**OASIS-3**	1.4K	T1w, T2w, FLAIR, ASL, DTI, rs-fMRI, PET	Y	42-95	Y	Diverse (mostly White)	USA	Open	Free	Direct	All
**OASIS-4**	663	Structural MRI	Y	21-94	Y	N	USA	Open	Free	Direct	Perp, DS
**AIBL**	1.1K	3T T1, amy-PET, FDG-PET	P	60-85	Y	Predom. White	Australia	Res.	Free	Reg.	DS, Gem, Cop
**NACC**	40K+	Variable MRI	N	50+	Y	Mixed (US)	USA	Res.	Free	App.	DS, Perp, Cop
**DLBS**	350	3T T1, fMRI, DTI	Y	20-89	Y	Mixed (US)	USA	Open	Free	Direct	DS
**BLSA**	1.5K	3T T1, DTI, fMRI, PET	P	20-100	Y	Mixed (US)	USA	Res.	Free	App.	DS
**UK Biobank**	50K+	3T T1, T2, fMRI, DTI	Y	40-70	Y	Mixed (UK)	UK	Res.	Free	App.	DS, Perp
**J-ADNI**	500	3T T1, PET	Y	50-85	Y	Asian	Japan	Res.	Free	App.	DS
**FHS**	5K	1.5T/3T T1, DTI	P	40-100	Y	Predom. White	USA	Res.	Free	App.	DS

**MIRIAD**	69	1.5T T1	Y	Elderly	N	Predom. White	UK	Open	Free	Direct	All ex. Gem

Continued on next page

Table 1 – continued

Dataset	N	Imaging Modalities	Prep	Age	Sex	Ethnicity	Location	Open Access	$	Access Type	LLMs
**EPAD**	1.4K	3D T1w, FLAIR, ASL, dMRI, rs-fMRI	Y	N/S	N	European	Europe	Open	Free	Direct	Perp, DS
**Kaggle AD MRI**	6.4K imgs	128x128 MRI	N	N/S	N	N	N/S	Open	Free	Direct	Perp
**PREVENT-AD**	350	3T T1, fMRI, PET	Y	55-80	Y	Predom. White	Canada	Res.	Free	App.	DS, Gem
**LEADS**	500	3T T1, PET	Y	40-64	Y	Mixed	USA	Res.	Free	App.	DS
**DIAN**	500	3T T1, PET	Y	20-50+	Y	Mixed	Global	Res.	Free	App.	DS, Gem, Cop
**HABS**	300	3T T1, PET	Y	60-90	Y	Predom. White	USA	Res.	Free	App.	DS
**WRAP**	1.5K	3T T1, DTI	P	40-65	Y	Predom. White	USA	Res.	Free	App.	DS
**BIOCARD**	300	3T T1, fMRI	Y	50-80	Y	Predom. White	USA	Res.	Free	App.	DS
**AddNeuro-Med**	500	1.5T T1, PET	Y	60-85	N	European	Europe	Res.	Free	App.	DS

**EMIF-AD**	1.3K	1.5T/3T T1, PET	Y	60-90	N	European	Europe	Res.	Free	App.	DS
**BrainLat**	780	3T MRI + EEG	Y	21-89	Y	Latin American	Latin America	Open	Free	Direct	Perp
**GAAIN**	Varies	Multi-modal	Varies	Varies	V	Varies	Global	Mixed	Varies	Varies	Perp, Gem, Cop
**ADDI**	Varies	Links to original datasets	Varies	Varies	V	Varies	Global	Yes	Free	Unified query	Cop, DS, Gem

Footnote - Abbreviations:

N = Not reported; N/S = Not Specified; Y = Yes; P = Partial; K = Thousand; Res. = Restricted; App. = Application; Reg. = Registration; Predom. = Predominantly; Amy = Amyloid; Ex. = Except;

AD = Alzheimer's Disease; ADDI = Alzheimer's Disease Data Initiative; ADNI = Alzheimer's Disease Neuroimaging Initiative; AIBL = Australian Imaging, Biomarkers & Lifestyle Study; BLSA = Baltimore Longitudinal Study of Aging; Cop = Microsoft Copilot; DIAN = Dominantly Inherited Alzheimer Network; DLBS = Dallas Lifespan Brain Study; DS = DeepSeek; DTI = Diffusion Tensor Imaging; EEG = Electroencephalography; EMIF-AD = European Medical Information Framework for Alzheimer's Disease; EPAD = European Prevention of Alzheimer's Dementia; FDG = Fluorodeoxyglucose; FHS = Framingham Heart Study; fMRI = functional Magnetic Resonance Imaging; GAAIN = Global Alzheimer's Association Interactive Network; Gem = Google Gemini; HABS = Harvard Aging Brain Study; J-ADNI = Japanese Alzheimer's Disease Neuroimaging Initiative; LEADS = Longitudinal Early-onset Alzheimer's Disease Study; LLM = Large Language Model; MIRIAD = Minimal Interval Resonance Imaging in Alzheimer's Disease; MRI = Magnetic Resonance Imaging; NACC = National Alzheimer's Coordinating Center; OASIS = Open Access Series of Imaging Studies; Perp = Perplexity AI; PET = Positron Emission Tomography; PREVENT-AD = Pre-symptomatic Evaluation of Novel or Experimental Treatments for Alzheimer's Disease; rs-fMRI = resting-state functional MRI; UK = United Kingdom; USA = United States of America; WRAP = Wisconsin Registry for Alzheimer's Prevention.
