# Supplementary material for: Geographic and demographic gaps in publicly available Alzheimer’s disease datasets: A large language model-based discovery and analysis
Source: Digit Health. 2026 Jul 21;12:20552076261470698. doi: 10.1177/20552076261470698 (PMC13389145; doi:10.1177/20552076261470698)
Supplement: Supplemental Material - Geographic and demographic gaps in publicly available Alzheimer’s disease datasets: A large language model-based discovery and analysis [file sj-pdf-3-dhj-10.1177_20552076261470698.pdf]

Table 1: Supplementary Table S2: Tabular Alzheimer's/Dementia Datasets Identified via LLM-Based Search (March–April 2025). This table includes datasets with tabular data types (clinical, cognitive, genetic, etc.) identified across five LLMs. Gender column is excluded; 'N' indicates Not reported. Abbreviations are provided in the footnote.

| Dataset | Description | N | Data Types | Prep | Age | Sex | Ethnicity | Location | Open Access | $ | Link | LLMs |
|---|---|---|---|---|---|---|---|---|---|---|---|---|
| **ADNI** | Longitudinal study: clinical, imaging, genetic, biochemical biomarkers | 2K+ | Clinical, MRI, PET, CSF, genetics | Y | 55-90 | Both | Mostly White | USA, Canada | Res. (app.) | Free | Link | C, DS, Perp, G |
| **PREVENT-AD** | Canadian presymptomatic AD cohort | 349 | Demo., cognitive, MRI, CSF, genetics | Y | N | Both | N | Canada | Partially open | Free | Link | C, G |
| **HABS** | Early AD markers via neuroimaging and cognitive tests | N | Clinical, neuropsych., MRI, PET | Y | N | Both | N | USA | Res. (app.) | Free | Link | C, G |
| **NIMH AD Genetics** | Genetic and phenotypic data for AD genetics | 1.4K | Genetic, clinical | Y | N | Both | N | USA | Res. | Free | Link | C, M |

**Table 1 – continued**

| Dataset | Description | N | Data Types | Prep | Age | Sex | Ethnicity | Location | Open Access | $ | Link | LLMs |
|---|---|---|---|---|---|---|---|---|---|---|---|---|
| **Kaggle AD Patient** | Demographic, lifestyle, medical, cognitive data | 2.1K | Demo., medical history, cognitive, lifestyle | Y | 60-90 | Both | Caucasian, Afr. Am., Asian, Other | N | Yes | Free | Link | C, G |
| **OASIS-3** | Longitudinal neuroimaging, clinical, cognitive data | 1.1K | MRI, PET, clinical | Y | N | Both | N | USA | Res. (app.) | Free | Link | C, DS, G |
| **AIBL** | Imaging, biomarkers, lifestyle factors | 1.1K | Clinical, imaging, genet-ics, lifestyle | Y | 60+ | Both | N | Australia | Partially open | Free | Link | C, DS, G |
| **SEA-AD** | Single-cell transcriptomic data from postmortem brains | N | Singlecell RNA seq. | Y | N | Both | N | USA | Yes | Free | Link | C, G |
| **NACC** | Large database of standardized clinical and neuropath. data | 40K+ | Clinical, cognitive, neuropath. | Y | 50+ | M/F (48/52%) | Mostly White (83%) | USA | Partially open | Free | Link | DS, Perp, G |

Continued on next page

**Table 1 – continued**

| Dataset | Description | N | Data Types | Prep | Age | Sex | Ethnicity | Location | Open Access | $ | Link | LLMs |
|---|---|---|---|---|---|---|---|---|---|---|---|---|
| **OASIS (Overall)** | Cross-sectional and longitudinal MRI, clinical, demo. data | 1K+ | MRI, clinical, cognitive | Y | 18-96 | M/F | Mostly White | USA | Yes | Free | Link | DS, G |
| **FHS** | Multigenerational cohort: cognitive, genetic, clinical data | 5K+ | Clinical, genetic, cognitive | Y | 40-100+ | M/F | Mostly White | USA | Partially open | Free | Link | DS, G |

Continued on next page

| Dataset | Description | N | Data Types | Prep | Age | Sex | Ethnicity | Location | Open Access | $ | Link | LLMs |
|---|---|---|---|---|---|---|---|---|---|---|---|---|
| **ROS/MAP** | Longitudinal clinical, cognitive, neuropath. data | 3K+ | Clinical, cognitive, neuropath. | Y | 65+ | M/F | Mostly White | USA | Partially open | Free | Link | DS, G |
| **DOD-ADNI** | ADNI extension focusing on veterans | 600+ | MRI, PET, blood biomarkers | Y | 55-90 | M/F | Diverse | USA | Partially open | Free | Link | DS, G |
| **AddNeuro-Med** | European cohort with MRI, blood biomarkers, clinical data | 500+ | MRI, clinical, biomarkers | Y | 65+ | M/F | Mostly White | Europe | Partially open | Free | Link | DS, G |

**Table 1 – continued**

| Dataset | Description | N | Data Types | Prep | Age | Sex | Ethnicity | Location | Open Access | $ | Link | LLMs |
|---|---|---|---|---|---|---|---|---|---|---|---|---|
| **EDSD** | DTI and clinical data in dementia | 500+ | DTI, clinical | Y | 50-85 | M/F | Mostly White | Europe | Partially open | Free | Link | DS, G |
| **ADPR** | Clinical, imaging, genetic data for predictive modeling | 1K+ | MRI, clinical, genetic | Y | 55-90 | M/F | Mostly White | Europe, USA | Partially open | Free | Link | DS, G |
| **LAADC** | Preclinical and early dementia stages | 1K | Clinical, MRI, genetic, cognitive | Y | Adults, elderly | Mixed | N | Oregon, USA | Yes | Free | Link | Perp |

Continued on next page

| Dataset | Description | N | Data Types | Prep | Age | Sex | Ethnicity | Location | Open Access | $ | Link | LLMs |
|---|---|---|---|---|---|---|---|---|---|---|---|---|
| **UK Biobank** | Large biobank: health, genetic, imaging, lifestyle data | 500K total | Clinical, imaging, genet-ics, lifestyle | Y | 40-69 | Mixed | Diverse UK | UK | Yes | Free | Link | Perp, G |
| **ADSP (NI-AGADS)** | Genetic and genomic data repository | Thousands | Genetic seq., phenotype | Y | Varies | Mixed | Diverse cohorts | US-based | Yes | Free | Link | Perp |

**Table 1 – continued**

| Dataset | Description | N | Data Types | Prep | Age | Sex | Ethnicity | Location | Open Access | $ | Link | LLMs |
|---|---|---|---|---|---|---|---|---|---|---|---|---|
| **AD Workbench** | Collection of 100+ datasets for AD and related dementias | Varies | Tabular | Y | Varies | Both | N | Global | Open access | Free | Link | M |
| **Mendeley AD** | Image classification of brain MRI for AD prediction | N | MRI | Y | N | Both | N | N | Open access | Free | Link | M |
| **OpenNeuro AD** | Platform for open neuroscience data including EEG | Varies | EEG, demo., MMSE | Y | 60s (mean) | Both | N | N | Open access | Free | Link | G |
| **Health Indicator (Alberta)** | Age-sex specific prevalence of dementia for Alberta | N | Demographics, prevalence | Y | N | Both | N | Alberta, Canada | Yes | Free | Link | N/A |

Continued on next page

| Dataset | Description | N | Data Types | Prep | Age | Sex | Ethnicity | Location | Open Access | $ | Link | LLMs |
|---|---|---|---|---|---|---|---|---|---|---|---|---|
| **CDC AD & Healthy Aging** | BRFSS surveys on aging, cognitive health | Varies | Survey data, health indicators | Y | Varies | Both | N | USA | Yes | Free | Link | N/A |

**Table 1 – continued**

| Dataset | Description | N | Data Types | Prep | Age | Sex | Ethnicity | Location | Open Access | $ | Link | LLMs |
|---|---|---|---|---|---|---|---|---|---|---|---|---|
| **BLSA** | Baltimore Longitudinal Study of Aging | 3K+ | Cognitive, physical, blood, urine, tissues | N | 20-100+ | M/F | Mostly White | USA | Res. | Free | Link | N/A |
| **CHARIOT-PRO** | Early Alzheimer's biomarker study | 1K+ | SocioDemo., health, cognition, lifestyle, biosamples | N | 60-85 | N | N | UK, Europe | Controlled access | Free | Link | N/A |
| **LEADS** | Longitudinal Early-onset AD Study | 500+ | Clinical, cognitive, imaging, biomarker, genetic | Y | 40-64 | N | N | USA | Controlled access | Free | Link | N/A |
| **DIAN** | Dominantly Inherited AD Network | 850 | Clinical, cognitive, genetic, blood, CSF | N | 18+ | Both | Mostly White | Global | Controlled access | Free | Link | N/A |

Continued on next page

**Table 1 – continued**

| | | | | | | | | | | | | |
|---|---|---|---|---|---|---|---|---|---|---|---|---|
| **PPMI** | Parkinson's Progression Markers Initiative | 1.5K+ | Clinical, imaging, biomarkers | Y | 50-85 | Both | Diverse | Global | Res. | Free | Link | N/A |
| **A4 Study** | Anti-amyloid treatment in asymptomatic AD | 3.2K | CSF, MRI, PET, longitudinal cognitive | N | 65-85 | Both | Diverse | Australia, Canada, Japan, USA | Controlled access | Free | Link | N/A |
| **WRAP** | Wisconsin Registry for Alzheimer's Prevention | 1.8K+ | Clinical, cognitive, survey, plasma, genomics, CSF, MRI, PET | Y | 55.5 (avg) | Both | Mostly White | USA | Controlled access | Free | Link | N/A |
| **ADCS** | Alzheimer's Disease Cooperative Study legacy trials | Varies | Clinical trials, cognitive | Y | Varies | Both | Diverse | USA | Controlled access | Free | Link | N/A |
| **NIA-LOAD** | Late-Onset AD Family Study | 2K+ | Genetic, clinical | Y | 60+ | Both | Mostly White | USA | Res. | Free | Link | N/A |

Continued on next page

**Table 1 – continued**

| Dataset | Description | N | Data Types | Prep | Age | Sex | Ethnicity | Location | Open Access | $ | Link | LLMs |
|---|---|---|---|---|---|---|---|---|---|---|---|---|
| **ACT** | Adult Changes in Thought Study | 7.5K+ | Medical records, EHR, cognitive, genet-ics, neuropath., MRI | Y | 65+ | N | Mostly White | USA | Controlled access | Free | Link | N/A |
| **HRS / ADAMS** | Health and Retirement Study / Aging, Demographics, and Memory Study | 20K+ (HRS), 856 (ADAMS) | Cognitive, clinical, proxy reports, genetics | Y | 50+ (HRS), 70+ (ADAMS) | Both | Diverse (oversampled Black/Hispanic) | USA | Controlled access | Free | Link | N/A |
| **NHATS** | National Health and Aging Trends Study | 12.4K | Cognitive, physical, functional, social, economic, end of life | N | 65+ | Both | Diverse (oversampled Black) | USA | Controlled access | Free | Link | N/A |

Continued on next page

| Dataset | Description | N | Data Types | Prep | Age | Sex | Ethnicity | Location | Open Access | $ | Link | LLMs |
|---|---|---|---|---|---|---|---|---|---|---|---|---|

**Table 1 – continued**

| | | | | | | | | | | | |
|---|---|---|---|---|---|---|---|---|---|---|---|
| **Health ABC** | Health, Aging and Body Composition Study | 3.1K | Genetic, imaging (DXA, MRI), physical activity, blood, cognitive | N | 70+ | Both | Black and White | USA | Controlled access | Free | Link | N/A |
| **EMIF-AD** | European Medical Information Framework for AD | Varies | Clinical, imaging, omics | N | N | N | N | UK, Europe | Controlled access | Free | Link | N/A |
| **MIRIAD** | Minimal Interval Resonance Imaging in AD | 70+ | MRI, demo-graphic, psychological | N | 55-85 | N | N | London | Fully open | Free | Link | N/A |
| **Synapse** | Shared dementia datasets (e.g., AddNeuroMed) | Varies | Clinical, omics, imaging | Y | Varies | Both | Varies | Varies | Varies | Free | Link | N/A |

**Table 1 – continued**

| | | | | | | | | | | | | |
|---|---|---|---|---|---|---|---|---|---|---|---|---|
| **GAAIN** | Global platform aggregating AD datasets | 743K+ | Clinical, cognitive, biomarker, imaging, genetics | Y | Varies | Both | Diverse | Global | Varies | Free | Link | N/A |

**Footnote - Abbreviations:**

N = Not reported; Y = Yes; K = Thousand; Res. = Restricted; App. = Application; Reg. = Registration; Demo. = Demographics; Neuropsych. = Neuropsychological; Afr. Am. = African American; SocioDemo. = Sociodemographic; Neuropath. = Neuropathology; Seq. = Sequencing;

AD = Alzheimer's Disease; ADAMS = Aging, Demographics, and Memory Study; ADCS = Alzheimer's Disease Cooperative Study; ADNI = Alzheimer's Disease Neuroimaging Initiative; ADPR = Alzheimer's Disease Prediction of Longitudinal Evolution; ADSP = Alzheimer's Disease Sequencing Project; AIBL = Australian Imaging, Biomarkers & Lifestyle Study; ACT = Adult Changes in Thought Study; BLSA = Baltimore Longitudinal Study of Aging; C = ChatGPT; CDC = Centers for Disease Control and Prevention; CSF = cerebrospinal fluid; DIAN = Dominantly Inherited Alzheimer Network; DOD = Department of Defense; DS = DeepSeek; DSS = NIAGADS Data Sharing System; DTI = diffusion tensor imaging; DXA = dual-energy X-ray absorptiometry; EDSD = European DTI Study on Dementia; EEG = electroencephalography; EHR = electronic health records; EMIF-AD = European Medical Information Framework for Alzheimer's Disease; FHS = Framingham Heart Study; G = Google Gemini; GAAIN = Global Alzheimer's Association Interactive Network; HABS = Harvard Aging Brain Study; Health ABC = Health, Aging and Body Composition Study; HRS = Health and Retirement Study; LAADC = Layton Aging & Alzheimer's Disease Center; LEADS = Longitudinal Early-onset Alzheimer's Disease Study; LLM = Large Language Model; M = Microsoft Copilot; MCI = mild cognitive impairment; MIRIAD = Minimal Interval Resonance Imaging in Alzheimer's Disease; MMSE = Mini-Mental State Examination; MRI = magnetic resonance imaging; NACC = National Alzheimer's Coordinating Center; NHATS = National Health and Aging Trends Study; NIA = National Institute on Aging; NIAGADS = National Institute on Aging Genetics of Alzheimer's Disease Data Storage Site; NIMH = National Institute of Mental Health; OASIS = Open Access Series of Imaging Studies; Perp = Perplexity AI; PET = positron emission tomography; PPMI = Parkinson's Progression Markers Initiative; PREVENT-AD = Pre-symptomatic Evaluation of Novel or Experimental Treatments for Alzheimer's Disease; ROS = Religious Orders Study; SEA-AD = Seattle Alzheimer's Disease Brain Cell Atlas; UK = United Kingdom; WRAP = Wisconsin Registry for Alzheimer's Prevention.
