# Supplementary material for: Geographic and demographic gaps in publicly available Alzheimer’s disease datasets: A large language model-based discovery and analysis
Source: Digit Health. 2026 Jul 21;12:20552076261470698. doi: 10.1177/20552076261470698 (PMC13389145; doi:10.1177/20552076261470698)
Supplement: Supplemental Material - Geographic and demographic gaps in publicly available Alzheimer’s disease datasets: A large language model-based discovery and analysis [file sj-pdf-4-dhj-10.1177_20552076261470698.pdf]

Table 1. Supplementary Table S3: Ethnicity Distribution Across Alzheimer's Datasets.

Dataset	Total	White	Asian	Black or AA	Multi Racial	Unknown	AI/ AN	NH/ PI	Hispanic/ Latino	Other
**OASIS**	2191	1846 (84.3%)	10 (0.5%)	335 (15.3%)	x	x	x	x	x	x
**NACC**	54631	45553 (83.4%)	1164 (2.1%)	5091 (9.3%)	1355 (2.5%)	1141 (2.1%)	262 (0.5%)	55 (0.1%)	x	x
**DLBS**	350	298 (85.1%)	11 (3.1%)	20 (5.7%)	x	x	4 (1.1%)	x	19 (5.4%)	19 (5.4%)
**BLSA**	1300	1045 (80.4%)	x	204 (15.7%)	x	51 (3.9%)	x	x	x	x
**J-ADNI**	537	x	537 (100.0%)	x	x	x	x	x	x	x
**HABS**	290	233 (80.3%)	6 (2.1%)	47 (16.2%)	x	4 (1.4%)	x	x	x	8 (2.8%)
**WRAP**	358	273 (76.3%)	1 (0.3%)	67 (18.7%)	5 (1.4%)	7 (2.0%)	4 (1.1%)	x	x	2 (0.6%)
**BIOCARD**	349	339 (97.1%)	x	x	x	x	x	x	x	10 (2.9%)
**A4 Study**	1169	1003 (85.8%)	22 (1.9%)	63 (5.4%)	x	x	x	x	52 (4.5%)	28 (2.4%)
**SEA-AD**	84	81 (96.4%)	3 (3.6%)	x	x	x	1 (1.2%)	x	x	3 (3.6%)
**BrainLat**	780	x	x	x	x	x	x	x	780 (100.0%)	x
**PPMI**	1400	1249 (89.2%)	15 (1.1%)	20 (1.4%)	32 (2.3%)	8 (0.6%)	1 (0.1%)	74 (5.3%)	x	x

Note: Values represent participant counts with percentages in parentheses. "x" denotes "Not Reported", indicating that the category was not collected or is not available. Totals may exceed 100% due to overlapping categories and rounding. AA: African American, AI/AN: American Indian/Alaska Native, NH/PI: Native Hawaiian/Pacific Islander.
